# Cost-Effectiveness of Community-based Human Immunodeficiency Virus Self-Testing in Blantyre, Malawi

**DOI:** 10.1093/cid/cix983

**Published:** 2017-11-09

**Authors:** Hendramoorthy Maheswaran, Aileen Clarke, Peter MacPherson, Felistas Kumwenda, David G Lalloo, Elizabeth L Corbett, Stavros Petrou

**Affiliations:** 1 Department of Public Health and Policy, University of Liverpool, United Kingdom; 2 Division of Health Sciences, University of Warwick Medical School, Coventry, United Kingdom; 3 Malawi-Liverpool-Wellcome Trust Clinical Research Programme, Blantyre, Malawi; 4 Department of Clinical Sciences, Liverpool School of Tropical Medicine, United Kingdom; 5 London School of Hygiene and Tropical Medicine, United Kingdom

**Keywords:** HIV, HIV self-testing, ART, cost-effectiveness, cost-utility

## Abstract

**Background:**

Human immunodeficiency virus self-testing (HIVST) is effective, with scale-up underway in sub-Saharan Africa. We assessed cost-effectiveness of adding HIVST to existing facility-based HIV testing and counseling (HTC) services. Both 2010 (initiate at CD4 <350 cells/μL) and 2015 (initiate all) World Health Organization (WHO) guidelines for antiretroviral treatment (ART) were considered.

**Methods:**

A microsimulation model was developed to evaluate cost-effectiveness, from both health provider and societal perspectives, of an HIVST service implemented in a cluster-randomized trial (CRT; ISRCTN02004005) in Malawi. Costs and health outcomes were evaluated over a 20-year time horizon, using a discount rate of 3%. Probabilistic sensitivity analysis was conducted to account for parameter uncertainty.

**Results:**

From the health provider perspective and 20-year time horizon, facility HTC using 2010 WHO ART guidelines was the least costly ($294.71 per person; 95% credible interval [CrI], 270.79–318.45) and least effective (11.64 quality-adjusted life-years [QALYs] per person; 95% CrI, 11.43–11.86) strategy. Compared with this strategy, the incremental cost-effectiveness ratio (ICER) for facility HTC using 2015 WHO ART guidelines was $226.85 (95% CrI, 198.79–284.35) per QALY gained. The strategy of facility HTC plus HIVST, using 2010 WHO ART guidelines, was extendedly dominated. The ICER for facility HTC plus HIVST, using 2015 WHO ART guidelines, was $253.90 (95% CrI, 201.71–342.02) per QALY gained compared with facility HTC and using 2015 WHO ART guidelines.

**Conclusions:**

HIVST may be cost-effective in a Malawian population with high HIV prevalence. HIVST is suited to an early HIV diagnosis and treatment strategy.

**Clinical Trials registration:**

ISRCTN02004005.

## Teaser

A cost-effectiveness analysis of community-based human immunodeficiency virus self-testing (HIVST) suggests that implementation is potentially cost effective in Malawi. The scale-up of HIVST is suited to countries with high HIV prevalence that target early diagnosis and initiation of antiretroviral treatment.

More than half of all people living with human immunodeficiency virus (PLHIV), new HIV infections, and HIV-related deaths are in eastern and southern Africa [1]. Despite intensive efforts to meet 90-90-90 Joint United Nations Programme on HIV/AIDS testing, treatment, and retention goals, nearly half of PLHIV remain unaware of their HIV status [2]. HIV testing and counselling (HTC) in health facilities is essential but remains underutilized [3]. Community-based HIV testing strategies have greater reach, but delivery of these services remains costly and difficult to sustain and can fail to offer satisfactory levels of privacy [4].

HIV self-testing (HIVST) resolves many of these issues by enabling individuals to perform and interpret their own HIV test result in private [2] and can be delivered to communities safely and at low cost by trained volunteers [5, 6]. HIVST has achieved high population HTC uptake, especially among men, and good rates of linkage into HIV treatment [5]. However, no formal economic evaluation has been undertaken to inform regional policy makers whether scaling up of self-testing offers efficient use of scarce resources.

Recently, a pragmatic cluster-randomized trial (CRT) was undertaken to investigate the impact of offering population-wide HIVST through community volunteers in Blantyre, Malawi (ISRCTN02004005) [5]. In this study, we undertook a cost-effectiveness analysis of this community-based HIVST intervention. We sought to use clinical effectiveness and economic data collected from participants of this trial [5, 7, 8], as well as data from secondary sources, and extrapolate the findings to the population level and over longer time horizons than observed in the trial. In addition, we explored the effects of changes in World Health Organization (WHO) and Malawian antiretroviral treatment (ART) initiation guidelines, which occurred after completion of the trial [9].

## METHODS

### Analytic Overview

We developed a microsimulation model to explore the impact of implementing HIVST in communities with high HIV prevalence and available facility-based HTC. The model simulates health provider and societal costs, health consequences of acquiring HIV infection, HIV disease progression, and initiation of ART. Simulating these costs and health consequences at the individual level has the advantage that parameters (eg, likelihood of accessing HIV testing) can reflect individual-level characteristics (eg, age, sex). The model drew heavily on evidence from the CRT [5].

### Human Immunodeficiency Virus Testing and Treatment Strategies

During the CRT (February 2012 to August 2014), Malawi used the 2010 WHO ART guidelines, with ART initiated if the patient had a CD4 count <350 cells/mm^3^, was WHO stage 3 or 4, or was pregnant or breastfeeding [10]. Since August 2016, Malawi has used the 2015 WHO ART guidelines with ART offered to all HIV-positive individuals [9]. We therefore evaluated 4 strategies. The base case was defined as availability of facility HTC, using 2010 ART guidelines. We compared this strategy to availability of facility HTC plus HIVST, using 2010 ART guidelines; availability of facility HTC, using 2015 ART guidelines; and availability of facility HTC plus HIVST, using 2015 ART guidelines. We did not consider the potential impact of other HIV prevention interventions.

### Decision-Analytic Model

**Figure 1.**
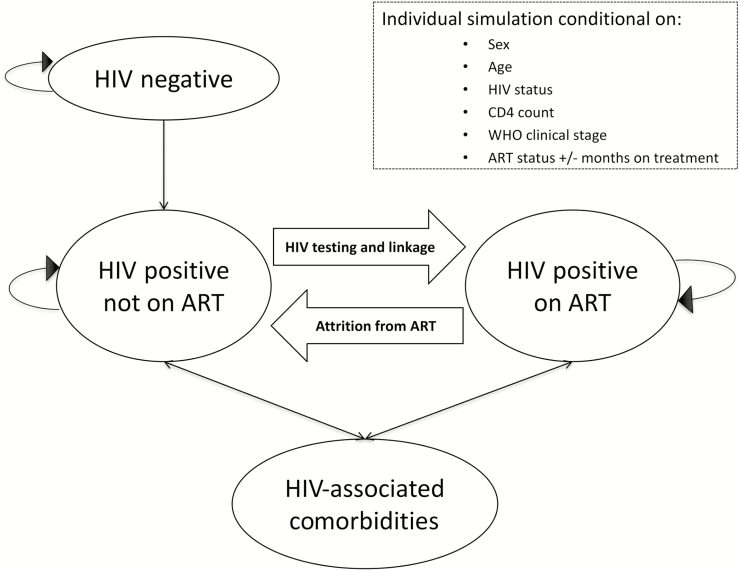
Overview of the microsimulation model. Abbreviations: ART, antiretroviral treatment; HIV, human immunodeficiency virus; WHO, World Health Organization.

Decision-analytic modeling used TreeAge Pro 2017 (TreeAge Software, Williamstown, Massachusetts). Figure 1 provides an overview of the model structure, which contained the following 4 health states: HIV negative, HIV positive and not on ART, HIV-associated comorbidities, and HIV positive and on ART. Every month, individuals transitioned through these health states. The model records and, in some cases, updates certain characteristics including sex, age, HIV status, CD4 count, WHO clinical stage, ART status, and months of ART received. These characteristics were used to estimate uptake of HIV testing, HIV incidence and prevalence, eligibility for ART initiation, risk of mortality, risk of HIV-associated comorbidities, and retention on ART.

### Characteristics of Individuals

Baseline population characteristics were estimated from the trial post-intervention survey in control clusters, showing 58% of participants were female; mean age was 30 years; HIV prevalence ranged from 2.3% in males aged 16–19 years to 28.6% in females aged 40–49 years; 2.1% were HIV positive with a CD4 count ≤50 cells/μL and 36.6% were HIV positive with a CD4 count >500 cells/μL (Table 1).

**Table 1.** Overview of Base-case Model Parameters

Parameter	Data	Source
Individual characteristics Age and sex demographics HIV prevalence CD4 counts in HIV positive	58% female; mean age 30 years 2.3%–28.6% (age and sex dependent) 36.6% CD4 count >500 cells/μL 23.4% CD4 count 351–500 cells/μL 22.8% CD4 count 201–350 cells/μL 15.2% CD4 count 51–200 cells/μL 2.1% CD4 count ≤50 cells/μL	Unpublished trial data (post intervention survey)
HIV testing and linkage into HIV care Annual uptake of facility HTC Annual uptake of HIVST Linkage after facility HTC Linkage after HIVST	14.7%–53.0% (age and sex dependent) 40.8%–99.9% (age and sex dependent) 50.7% (95% CI, 44.9–56.6) 41.7% (95% CI, 38.8–44.4)	Unpublished trial data [5] [11] [5]
HIV incidence and mortality HIV incidence Non-HIV mortality HIV mortality, not on ART HIV mortality, on ART	0.66–6.49 per 100 person-years (age and sex dependent) Malawi life tables (age and sex dependent) 0.6–69.5 per 100 person-years (CD4 count dependent) 1.4–14.0 per 100 person-years (CD4 count dependent)	[22] [20] [18] [19]
Mean change in CD4 count Not on ART On ART	Decreases 4.0–5.7 cells/month (CD4 count dependent) Increases 1.3 cells/week (95% CI, 1.1–1.5)	[16, 25–27] [19]
HIV treatment outcomes Pre-ART returning for ART assessment in 6 months Retention on ART	57.1% (95% CI, 56.0, 58.0) 0–6 months: 86.1% (95% CI, 84.6, 87.4) 7–12 months: 80.2% (95% CI, 78.0, 82.4) 13–24 months: 76.1% (95% CI, 72.4, 79.7) >24 months: 72.3% (95% CI, 67.4, 76.9)	[12–17] [28]
HIV-associated illnesses	CD4 count dependent	[18, 27]

Abbreviations: ART, antiretroviral treatment; CI, confidence interval; HIV, human immunodeficiency virus; HIVST, human immunodeficiency virus self-testing; HTC, human immunodeficiency virus testing and counselling.

### Human Immunodeficiency Virus (HIV) Testing and Linkage Into HIV Care

Trial data were used to derive probabilities for accessing each testing modality by sex and age [5]. Individuals who tested HIV negative did not retest for 1 year. In the trial, the HIVST service was provided independently of existing facility-based HTC. Therefore, we assumed mutually exclusive probabilities for accessing HIV testing modalities. Those who tested HIV positive through HIVST incurred an additional cost for facility-based confirmatory HIV testing. A cohort study conducted before introduction of HIVST provided estimates of linkage into HIV treatment after facility HTC [11]. Linkage into HIV treatment after HIVST was based on trial findings [5]. For strategies that included 2010 WHO ART guidelines, data from the literature were used to model the likelihood of those not eligible for ART returning for repeat assessment for ART initiation [12–17].

### Transition Probabilities

Death occurred from HIV-related [18, 19] and unrelated causes, with Malawian-specific age and sex mortality rates used to model HIV-unrelated mortality [20]. HIV-negative individuals were at risk of acquiring HIV infection. As the model did not allow for interaction between individuals modeled [21], we assumed HIV incidence varied by age and sex but was otherwise constant and used estimates from a South African study with ART coverage comparable to that in Malawi [22].

For HIV-positive individuals, a CD4 count was assigned on entry into the model or when infected with HIV. HIV-positive individuals’ CD4 counts decreased when not receiving ART and increased when receiving ART [23], with rates of change estimated from previous studies [16, 19, 24–27]. Modeled CD4 counts determined individual eligibility to start ART and likelihood of HIV-associated comorbidity or death. The model simulated progression to WHO clinical stages 3 or 4 [18] to account for ART eligibility under 2010 WHO guidelines.

The model was parameterized to account for time-varying rates of ART discontinuation [28]. If treatment was discontinued, individuals returned to the “HIV-positive not on ART” health state. The model did not account for ART failure or HIV viral load monitoring, as this was not offered at the time of the trial. ART failure may require switching to more expensive second-line ART regimens; however, this remains uncommon in the region [29].

For HIV-associated comorbidities, we only considered the costs and impact on health-related quality of life (HRQoL) arising from hospitalization [30]. For HIV-positive individuals not on ART, we multiplied the risk of experiencing these HIV-associated comorbidities [18, 27] by the risk of hospitalization [18]. For HIV-positive individuals on ART, we additionally multiplied these risks by the relative reduction in hospitalization attributable to ART [31]. We assumed that HIV-positive individuals on ART who were hospitalized continued to receive ART. We assumed individuals who experienced these comorbidities would undergo HIV testing with a similar likelihood of linking into HIV treatment as after facility HTC. Additional information about the modeling approach (Supplementary material Appendix A), model parameter synthesis processes (Supplementary material Appendix B), and model external validation procedures (Supplementary material Appendix C) are provided.

### Costs

The direct health provider and societal costs of facility HTC, HIVST, assessment for ART eligibility, and ART were all derived from primary costing studies that recruited participants from the CRT [7, 8]. The costs associated with different HIV-associated comorbidities were derived from primary costing of adult medical admissions to the main public hospital in Blantyre [30]. Costs were adjusted to reflect the 1-month cycle length used in the model. Societal costs incorporated estimates of direct health provider costs, direct nonmedical costs, and indirect costs. Table 2 shows the cost parameters in 2014 US dollars.

**Table 2.** Health Provider and Societal Costs for Model

Cost Parameter	2014 US Dollars						Distribution
	Health Provider Costs			Societal Costs			
	Base Case	Low	High	Base Case	Low	High	
Facility-based HTC episode	8.90	7.53	10.57	10.68	9.91	11.45	
HIV self-testing episode	8.78	7.78	10.46	8.85	7.97	9.72	
Assessment for ART eligibility for all clients	22.27	21.32	23.21	25.46	24.14	26.79	
Annual cost of ART for facility HTC clients	168.65	164.69	172.62	181.91	175.38	188.45	
Annual cost of ART for facility HIVST clients	164.66	156.41	172.90	179.38	164.29	194.46	Gamma
Cost of hospital admission for severe HIV-associated illness							
Acute diarrhea	300.97	134.37	467.56	481.56	190.30	772.82	
Chronic diarrhea	233.06	93.84	372.28	372.28	114.42	407.39	
Esophageal candidiasis	153.08	69.92	236.24	236.24	65.30	292.59	
Invasive bacterial diseases	223.45	199.68	247.21	247.21	229.39	291.01	
Pulmonary tuberculosis	437.68	339.02	536.33	536.33	441.79	716.81	
Extrapulmonary tuberculosis	494.68	394.83	594.53	594.53	526.86	1014.00	
Malaria	199.63	106.55	292.72	292.72	69.06	647.84	
Malignancy (Kaposi’s sarcoma/Lymphoma)	242.92	195.53	290.31	290.31	244.64	389.41	
Pneumocystis Jivorecii pneumonia	325.56	268.15	382.97	382.97	294.62	495.67	
Cryptococcal meningitis	846.24	651.05	1041.44	1041.44	760.87	1194.62	

Abbreviations: ART, antiretroviral treatment; HIV, human immunodeficiency virus; HIVST, human immunodeficiency virus self-testing; HTC, human immunodeficiency virus testing and counselling.

### Health-related Quality of Life

The primary health outcome was quality-adjusted life-years (QALYs), estimated by multiplying health utility scores assigned to the different health states in the model by the time spent in each health state and summing across health states [32]. Utility scores varied by HIV status. For HIV-positive individuals, utility scores decreased as CD4 count decreased and following HIV-associated comorbidity [33, 34]. Utility scores for all health states were derived from primary economic studies in Blantyre that recruited participants from the CRT [7, 8] or from adult medical admissions [30]. In these studies the Chichewa version of the EuroQoL EQ-5D-3L [35] was used to assess participants’ HRQoL. The EQ-5D utility scores for the health states were derived using the Zimbabwean [36] EQ-5D tariff set (Table 3).

**Table 3.** EQ-5D Utility Scores for Health States: Zimbabwean and UK Tariff

Utility Parameter	EQ-5D Utility Score			Distribution
	Base Case	Low	High	
HIV negative	1.000	1.000	1.000	Beta
HIV positive not on ART				
CD4 >200 cells/μL	0.878	0.802	0.954	
CD4 51–200 cells/μL	0.840	0.762	0.917	
CD4 count ≤50 cells/μL	0.654	0.558	0.749	
Increase over first year on ART for facility HTC clients	0.129	0.107	0.150	
Increase over first year on ART for HIVST clients	0.139	0.087	0.192	
Hospital admission for severe HIV associated illness				
Acute diarrhea	0.367	0.143	0.590	
Chronic diarrhea	0.476	0.316	0.636	
Esophageal candidiasis	0.349	0.170	0.529	
Invasive bacterial diseases	0.499	0.457	0.541	
Pulmonary tuberculosis	0.429	0.349	0.509	
Extrapulmonary tuberculosis	0.389	0.296	0.481	
Malaria	0.567	0.412	0.721	
Malignancy (Kaposi’s sarcoma/Lymphoma)	0.420	0.320	0.521	
Pneumocystis Jivorecii pneumonia	0.559	0.398	0.719	
Cryptococcal meningitis	0.478	0.386	0.569	

Abbreviations: ART, antiretroviral treatment; HIV, human immunodeficiency virus; HIVST, human immunodeficiency virus self-testing; HTC, human immunodeficiency virus testing and counselling.

### Cost-Effectiveness Analysis

The model was used to project the costs and QALYs for each testing/ART strategy. A time horizon of 20 years rather than the standard lifetime horizon [32] was used, given likely changes in HIV incidence and testing and treatment strategies over time. Scenario analyses included alternative time horizons of 10 and 40 years.

Probabilistic sensitivity analysis was used to address parameter uncertainty. The beta distribution was fitted to transition probabilities and health state utilities and to the gamma distribution for costs [37]. For each strategy, we ran 5000 model runs, randomly selecting a value for each parameter from its distribution. For each model run, we estimated total costs and QALYs for a sample of 5000 individuals.

We report mean discounted costs and QALYs per person across these simulations for each testing/ART strategy. We estimated the mean incremental cost and incremental QALYs by comparing the least-costly and least-effective strategy to the next least-costly and least-effective strategy. The incremental cost-effectiveness ratio (ICER) for respective comparators was calculated by dividing incremental costs by incremental QALYs gained. We excluded strategies that were dominated, that is, less effective and more costly, or extendedly dominated, where the ICER for the strategy is higher than a more effective strategy. All results are presented with 95% credible intervals (CrIs). This interval represents the 2.5th and 97.5th percentiles from the distribution of results from all simulations. Separate analyses were undertaken from health provider and societal perspectives [32]. Costs are represented in 2014 US and international dollars, and a discount rate of 3% was applied to both costs and health effects.

We compared estimated ICERs against increasing cost-effectiveness thresholds as follows: $0/QALY, $250/QALY, $500/QALY, and $750/QALY. For each testing/ART strategy, we present the probability of cost effectiveness at these thresholds. This probability represents the proportion of all simulations where the estimated ICER was below the specified cost-effectiveness threshold [32]. Because we compared multiple strategies and because decision makers may have different cost-effectiveness thresholds, we also present cost-effectiveness acceptability frontiers (CEAFs) [38] to show which strategy is optimal at increasing cost-effectiveness thresholds. We undertook a series of deterministic sensitivity analyses, using the point estimate for all parameters except the one being explored, to evaluate the impact on the ICER. We estimated the ICERs across the plausible ranges for the parameter of interest and present findings in a tornado plot.

## RESULTS

### Health Provider Costs

Over a 20-year time horizon and health provider perspective, availability of facility HTC and using 2010 WHO ART guidelines was the least costly strategy ($294.71 per person; 95% CrI, 270.79–318.45; Table 4). The next least costly strategy was facility HTC and using 2015 WHO ART guidelines ($336.13 per person; 95% CrI, 313.35–358.64). The two strategies of facility HTC plus HIVST, using either the 2010 or 2015 WHO ART guidelines, had higher mean discounted costs of $380.27 (95% CrI, 355.08–404.54) and $438.79 (95% CrI, 416.75–461.12) per person, respectively.

**Table 4.** Cost-Effectiveness Findings from Primary Analysis and 20-Year Time Horizon

Perspective	HIV Testing Strategy	ART Initiation Guideline	Discounted Mean Costs and QALYs per Person				Incremental Cost-Effectiveness Ratio	Probability Cost-Effective at Cost-Effectiveness Threshold^b^ (2014 US$ per QALY)			
			2014 US$		QALYs						
			Mean Cost (95% CrI ^a^)	Incremental Cost (95% CrI ^a^)	Mean Effectiveness (95% CrI ^a^)	Incremental Effectiveness (95% CrI ^a^)	2014 US$ per QALY (95% CrI ^a^)	0	250	500	750
Health provider	Facility HTC	2010 WHO ART	294.71 (270.79, 318.45)	-	11.64 (11.43, 11.86)	-	-	1.000	0.128	0	0
	Facility HTC	2015 WHO ART	336.13 (313.35, 358.64)	41.42 (29.86, 55.64)	11.82 (11.62, 12.03)	0.18 (0.12, 0.25)	226.85 (198.79, 284.35)	0	0.362	0.001	0
	Facility HTC and HIVST	2010 WHO ART	380.27 (355.08, 404.54)	-	11.99 (11.80, 12.18)	-	ED^c^	0	0.207	0	0
	Facility HTC and HIVST	2015 WHO ART	438.79 (416.75, 461.12)	102.66 (85.45, 120.04)	12.23 (12.06, 12.40)	0.40 (0.28, 0.53)	253.90 (201.71, 342.02)	0	0.303	0.999	1.000
Societal	Facility HTC	2010 WHO ART	334.70 (306.45, 363.54)	-	11.64 (11.43, 11.86)	-	-	1.000	0.178	0	0
	Facility HTC	2015 WHO ART	377.67 (351.29, 405.16)	42.98 (30.33, 58.84)	11.82 (11.62, 12.03)	0.18 (0.12, 0.25)	234.69 (198.76, 297.52)	0	0.368	0.003	0
	Facility HTC and HIVST	2010 WHO ART	422.82 (392.19, 452.10)	-	11.99 (11.80, 12.18)	-	ED^c^	0	0.238	0.001	0
	Facility HTC and HIVST	2015 WHO ART	484.16 (456.30, 512.96)	106.49 (84.90, 128.67)	12.23 (12.06, 12.40)	0.40 (0.28, 0.53)	262.68 (203.75, 363.20)	0	0.215	0.996	1.000

2010 WHO ART initiation guidelines: CD4 count <350 cells/mm^3^ or WHO stage 3 or 4. 2015 WHO ART initiation guidelines: start ART irrespective of CD4 count or WHO stage.

Abbreviations: ART, antiretroviral treatment; CrI, credible interval; ED, extendedly dominated; HIV, human immunodeficiency virus; HIVST, human immunodeficiency virus self-testing; HTC, human immunodeficiency virus testing and counselling; QALY, quality-adjusted life-year; WHO, World Health Organization.

a 95% CrI represents the 2.5th and 97.5th percentile from the distribution of results from all the simulations.

b Probability represents the proportion of all simulations where the estimated incremental cost-effectiveness ratio (ICER) was below the specified cost-effectiveness threshold. Total may not add up to 1.0 as for some simulations; no single scenario was found to be the most cost-effective at given cost-effectiveness threshold.

c Extended dominance: the ICER for this strategy was higher than the next more effective strategy.

### Societal Costs

Over a 20-year time horizon and societal perspective, facility HTC and using 2010 WHO ART guidelines was the least costly strategy ($334.70 per person; 95% CrI, 306.45–363.54). The next least costly strategy was facility HTC and using 2015 WHO ART guidelines ($377.67 per person; 95% CrI, 351.29–405.16). The two strategies of facility HTC plus HIVST, using either the 2010 or 2015 WHO ART guidelines, had higher mean discounted societal costs of $422.82 (95% CrI, 392.19–452.10) and $484.16 (95% CrI, 456.30–512.96) per person, respectively.

### Health Outcomes

Over a 20-year time horizon, facility HTC and using 2010 WHO ART guidelines was the least effective strategy (11.64 QALYs per person; 95% CrI, 11.43–11.86). The next least effective strategy was facility HTC and using 2015 WHO ART guidelines (11.82 QALYs per person; 95% CrI, 11.62–12.03). Facility HTC plus HIVST, using either the 2010 or 2015 WHO ART guidelines, was more effective, generating 11.99 (95% CrI, 11.80–12.18) and 12.23 (95% CrI, 12.06–12.40) QALYs per person, respectively.

### Cost-Effectiveness Analyses

From the health provider perspective and 20-year time horizon, the ICER for facility HTC and using 2015 WHO ART guidelines was $226.85 (95% CrI, 198.79–284.35) per QALY gained compared with facility HTC and using 2010 WHO ART guidelines (Table 4). The strategy of facility HTC plus HIVST and using 2010 WHO ART guidelines was extendedly dominated. The ICER for facility HTC plus HIVST and using 2015 WHO ART guidelines was $253.90 (95% CrI, 201.71–342.02) per QALY gained compared with facility HTC and using 2010 WHO ART guidelines.

From the societal perspective and 20-year time horizon, the ICER for facility HTC plus HIVST and using 2015 WHO ART guidelines was $234.69 (95% CrI, 198.76–297.52) per QALY gained compared with facility HTC and using 2010 WHO ART guidelines. The strategy of facility HTC plus HIVST and using 2010 WHO ART guidelines was extendedly dominated. The ICER for facility HTC plus HIVST and using 2015 WHO ART guidelines was $262.68 (95% CrI, 203.75–363.20) per QALY gained compared with facility HTC plus HIVST and using 2010 WHO ART guidelines. Supplementary material Appendix D shows the findings when the costs were estimated in 2014 international dollars.

**Figure 2.**
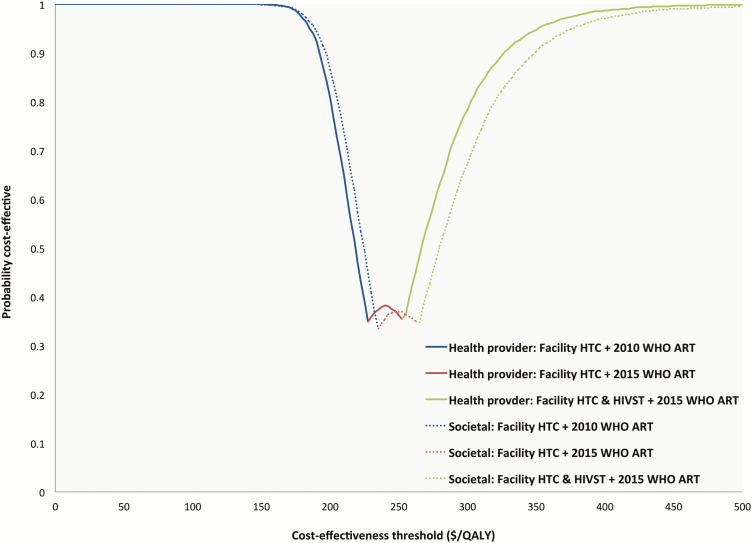
Cost-effectiveness acceptability frontier showing optimal strategy at increasing cost-effectiveness threshold values for gain in quality-adjusted life-year. 2010 World Health Organization (WHO) ART initiation guidelines: CD4 count <350 cells/mm^3^ or WHO stage 3 or 4. 2015 WHO ART initiation guidelines: start ART irrespective of CD4 count or WHO stage. Abbreviations: ART, antiretroviral treatment; HIVST, human immunodeficiency virus self-testing; HTC, human immunodeficiency virus testing and counselling; QALY, quality-adjusted life-year; WHO, World Health Organization.

Table 4 shows the probability that each strategy is cost effective at cost-effectiveness thresholds of $0/QALY, $250/QALY, $500/QALY, and $750/QALY. Figure 2 shows the CEAF for the optimal strategies across increasing cost-effectiveness threshold values. Up to a threshold value of approximately $200, the strategy of facility HTC and using 2010 WHO ART guidelines remained optimal in cost-effectiveness terms. At a cost-effectiveness threshold of $250/QALY, the strategy of facility HTC and using 2015 WHO ART guidelines was optimal. At threshold values greater than approximately $270, facility HTC plus HIVST and using 2015 WHO ART guidelines was the optimal strategy.

### Scenario Analyses

Over both the 10- and 40-year time horizons, the strategy of facility HTC plus HIVST and using 2015 WHO ART guidelines remained optimal at cost-effectiveness thresholds greater than $500 per QALY (Table 5).

**Table 5.** Cost-Effectiveness Findings from the Health Provider Perspective Over Different Time Horizons

Time Horizon	Human Immunodeficiency Virus Testing Strategy	ART Initiation Guideline	Discounted Mean Costs and QALYs per Person				Incremental Cost-Effectiveness Ratio	Probability Cost-Effective at Cost-Effectiveness Threshold^b^ (US$ per QALY)			
			2014 US$		QALYs						
			Mean Cost (^a^95% CrI ^a^)	Incremental Cost (95% CrI ^a^)	Mean Effectiveness (95% CrI ^a^)	Incremental Effectiveness (95% CrI ^a^)	2014 US$ per QALY (95% CrI ^a^)	0	250	500	750
10 years	Facility HTC	2010 WHO ART	157.77 (142.53, 171.95)	-	7.45 (7.35, 7.55)	-	-	1.000	0.998	0.031	0
	Facility HTC	2015 WHO ART	184.86 (172.07, 197.65)	27.09 (19.99, 36.41)	7.52 (7.42, 7.61)	0.07 (0.04, 0.10)	389.43 (311.05, 520.51)	0	0	0.211	0.019
	Facility HTC and HIVST	2010 WHO ART	206.82 (190.24, 221.96)	-	7.56 (7.47, 7.65)	-	ED^c^	0	0.002	0.054	0.002
	Facility HTC and HIVST	2015 WHO ART	247.64 (234.64, 260.74)	62.78 (53.20, 72.58)	7.67 (7.58, 7.74)	0.15 (0.09, 0.20)	430.47 (323.11, 645.72)	0	0	0.704	0.979
20 years	Facility HTC	2010 WHO ART	294.71 (270.79, 318.45)	-	11.64 (11.43, 11.86)	-	-	1.000	0.128	0	0
	Facility HTC	2015 WHO ART	336.13 (313.35, 358.64)	41.42 (29.86, 55.64)	11.82 (11.62, 12.03)	0.18 (0.12, 0.25)	226.85 (198.79, 284.35)	0	0.362	0.001	0
	Facility HTC and HIVST	2010 WHO ART	380.27 (355.08, 404.54)	-	11.99 (11.80, 12.18)	-	ED^c^	0	0.207	0	0
	Facility HTC and HIVST	2015 WHO ART	438.79 (416.75, 461.12)	58.52 (44.32, 76.69)	12.23 (12.06, 12.40)	0.24 (0.16, 0.32)	247.92 (207.60, 312.97)	0	0.303	0.999	1.000
40 years	Facility HTC	2010 WHO ART	408.07 (372.67, 445.05)	-	15.13 (14.72, 15.55)	-	-	1.000	0	0	0
	Facility HTC	2015 WHO ART	461.25 (424.82, 498.46)	-	15.46 (15.05, 15.88)	-	ED^c^	0	0.007	0	0
	Facility HTC and HIVST	2010 WHO ART	530.83 (495.99, 565.74)	122.77 (99.18, 146.04)	15.92 (15.64, 16.28)	0.79 (0.55, 1.03)	155.58 (127.21, 204.37)	0	0.033	0	0
	Facility HTC and HIVST	2015 WHO ART	602.34 (569.01, 635.77)	71.51 (51.89, 95.18)	16.32 (15.99, 16.66)	0.41 (0.24, 0.59)	175.77 (146.77, 236.34)	0	0.960	1.000	1.000

2010 WHO ART initiation guidelines: CD4 count < 350 cells/mm^3^ or WHO stage 3 or 4. 2015 WHO ART initiation guidelines: start ART irrespective of CD4 count or WHO stage.

Abbreviations: ART, antiretroviral treatment; CrI, credible interval; ED, extendedly dominated; HIVST, human immunodeficiency virus self-testing; HTC, human immunodeficiency virus testing and counselling; QALY, quality-adjusted life-year; WHO, World Health Organization.

a 95% CrI represents the 2.5th and 97.5th percentile from the distribution of results from all the simulations.

b Probability represents the proportion of all simulations where the estimated incremental cost-effectiveness ratio (ICER) was below the specified cost-effectiveness threshold. Total may not add up to 1.0 as for some simulations; no single scenario was found to be most cost-effective at given cost-effectiveness threshold.

c Extended dominance: the ICER for this strategy is higher than the next more effective strategy.

### Deterministic Sensitivity Analyses

**Figure 3.**
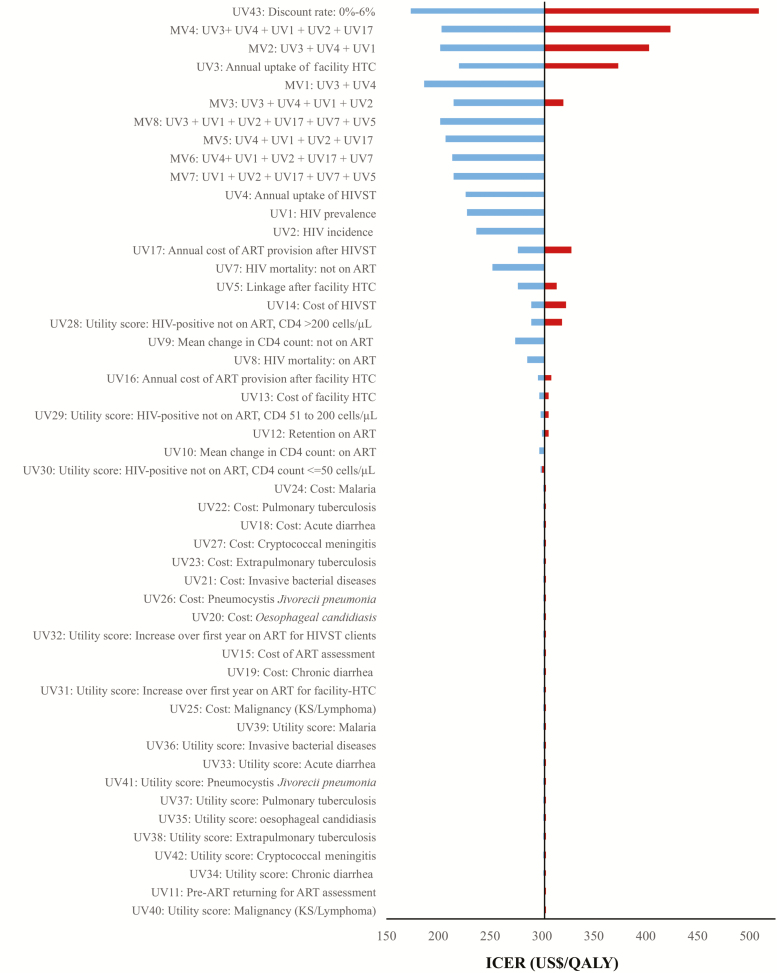
Tornado diagram showing findings from deterministic sensitivity analysis. Abbreviations: ART, antiretroviral treatment; HIV, human immunodeficiency virus; HIVST, human immunodeficiency virus self-testing; HTC, human immunodeficiency virus testing and counselling; ICER, incremental cost-effectiveness ratio; KS, Kaposi’s sarcoma; MV, multivariate sensitivity analysis; QALY, quality-adjusted life-year; UV, univariate/1-way sensitivity analysis.

Figure 3 shows a tornado plot from the deterministic sensitivity analysis that compares the strategy of facility HTC plus HIVST and using 2015 WHO ART guidelines to the strategy of facility HTC and using 2015 WHO ART guidelines. The uptake of facility-based HIV testing and HIVST, cost of HIVST episode, and the HIV prevalence and incidence in the population had the greatest impact on the ICER. Supplementary material Appendix E provides more detail on the findings from the deterministic sensitivity analysis.

## DISCUSSION

In this study, we undertook an economic evaluation of a community-based HIV self-testing service in Blantyre, Malawi, and estimated cost effectiveness, taking into account recent changes in the guidelines for when individuals should start ART. Overall, we found that providing community-based HIVST and using the 2015 WHO ART guidelines was the optimal strategy at cost-effective thresholds greater than $270/QALY. The gross domestic product in Malawi is approximately $250 per capita. The finding that delivery of HIV testing closer to people’s homes is cost effective is not new [39]; however, to our knowledge, this is the first evaluation of HIVST strategies to use robust data from a large CRT combined with primary economic studies.

Adopting the 2015 WHO ART guidelines or implementing HIVST will result in higher healthcare costs. In Malawi, adopting the 2015 WHO ART guidelines would cost healthcare providers an additional $41 per capita over the next 20 years and would equate to a 14% increase in HIV testing and treatment expenditures. Adopting both strategies would cost $144 per capita over the next 20 years and a 49% increase in HIV testing and treatment expenditure. However, implementation would have a synergistic effect, resulting in the greatest health gains. Uptake of HIV testing remains suboptimal in the region [2], with HIV-positive individuals with advanced HIV disease still only accessing HIV treatment services [40]. Implementing HIVST may be necessary to achieve the hoped-for health benefits from universal access to ART but needs to be balanced against local budgetary constraints and whether investment in other HIV and non-HIV interventions offers better value for the money.

We previously estimated the cost per individual tested through HIVST to be comparable to facility-based HTC [7]. The cost of HIVST kits is currently 8 times that of the rapid finger-prick test kits used in health facilities. We found the cost of an HIVST episode and ART provision to be important drivers of cost effectiveness. If the cost of an HIVST episode were lower and achievable if the cost of an HIVST kit fell from current estimates of $4 per kit and if the cost of ART provision were lower through lower ART drug costs, implementation of HIVST and adoption of the 2015 WHO ART guidelines would be seen as more affordable by policy makers in the region.

There are several limitations to our study. First, our analysis does not consider the impact of HIV transmission. In comparison to the base case, the 3 other strategies examined resulted in a net gain in QALYs as well as increased numbers on ART. As the number of HIV infections averted depends on ART coverage among HIV-positive individuals, considering HIV transmission is likely to have led to lower ICER estimates; therefore, our findings represent conservative estimates. Second, we did not consider the impact of individuals failing ART. At the time of the trial and of health economic studies, HIV viral load monitoring was not routinely available in Malawi and only 3% of HIV-positive individuals in the region had switched to second-line ART regimens [29]. However, as all strategies examined result in more HIV-positive individuals taking ART, the need for HIV viral load monitoring and costlier second-line ART regimens will increase. This is likely to lead to less favorable ICERs than those estimated. Finally, we only considered the impact of HIV-associated illnesses that required hospitalizations and did not consider other illnesses that are managed in the community or at primary health clinics. Again, as the costlier strategies result in earlier initiation of ART, had we considered these additional health sequelae, the ICER estimates would likely have been lower.

Achievement of high coverage of ART is essential to eliminating the HIV epidemic in sub-Saharan Africa but will require substantial increases in rates of HIV testing. HIVST is popular and can have a major impact on population coverage of HIV testing, with relatively little input from trained health professionals [5, 6]. We found implementation HIVST to be potentially cost effective. Notably, our model suggests that the transition from restricted ART availability to the 2015 WHO ART guidelines of immediate offer of ART irrespective of CD4 count combines favorably with HIVST. Without effective community HIV testing programs, the health benefits of universal access to ART are limited by the inability to detect early HIV efficiently under facility-only testing strategies. HIVST is therefore suited to an early HIV diagnosis and treatment strategy.

## Supplementary Data

Supplementary materials are available at *Clinical Infectious Diseases* online. Consisting of data provided by the authors to benefit the reader, the posted materials are not copyedited and are the sole responsibility of the authors, so questions or comments should be addressed to the corresponding author.

Supplementary Appendix

## Notes

***Author contributions.*** H. M. conceived and designed the study, conducted cost and statistical analyses, and drafted the manuscript. S. P., A. C., and E. L. C. supported design of the study and data collection tools. All authors interpreted the data, prepared the report, and approved the final version.

***Acknowledgements.*** We thank the community members who participated in the study and the patients and staff at Ndirande Health Centre, Chilomoni Health Centre, and Queen Elizabeth Central Hospital in Blantyre. We are grateful for all the staff at the Blantyre District Health Office and the HIV Department of the Ministry of Health of Malawi for aiding with the costing work.

***Disclaimer.*** This paper presents independent research, and the views expressed are those of the author(s) and not necessarily those of the Wellcome Trust, the National Health Service, the National Institute for Health Research (NIHR) or the Department of Health.

***Financial support.*** H. M. was supported by the Wellcome Trust (grant WT097973). E. L. C. was supported by the Wellcome Trust (grant WT091769). A. C. is supported by the NIHR Collaborations for Leadership in Applied Health Research and Care (CLAHRC) West Midlands initiative.

***Potential conflicts of interest***. All authors: No reported conflicts. All authors have submitted the ICMJE Form for Disclosure of Potential Conflicts of Interest. Conflicts that the editors consider relevant to the content of the manuscript have been disclosed.
