# Supplementary material for: Cost-Effectiveness of Community-based Human Immunodeficiency Virus Self-Testing in Blantyre, Malawi
Source: Clin Infect Dis. 2017 Nov 9;66(8):1211–21. doi: 10.1093/cid/cix983 (PMC5889018; doi:10.1093/cid/cix983)
Supplement: Supplementary Appendix [file cix983_suppl_supplementary_appendix.docx]

**Online Appendix**

**Maheswaran H, Clarke A et al. Cost-effectiveness of community-based HIV self-testing in Malawi**

Appendix A: Overview of modeling approach

Appendix B: Model parameter synthesis

Appendix C: Model validation

Appendix D: Primary cost-effectiveness analysis in International Dollars

Appendix E: Deterministic sensitivity analysis

**Appendix A: Overview of modeling approach**

An individual-level simulation model (SIM) was developed to simulate the impact of implementing different combinations of HIV testing services in a community with high HIV prevalence. ISMs are sometimes referred as Monte Carlo simulation or microsimulation [1-3]. The model considers the impact of acquiring HIV infection, HIV disease progression and initiation of anti-retroviral treatment.

Individual simulation models simulate the transition of an individual through the model as they accumulate costs, and record their health outcomes. The model then repeats the analysis for a large enough number of individuals to estimate the mean costs and health outcomes. As the model simulates an individual it can vary its parameters based on the characteristics on the individual [2, 3]. For example, this allows us to vary the likelihood of an individual accessing facility-based HIV testing and counselling (HTC) or HIV self-testing (HIVST) based on their sex and age.

Additionally, when developing the model we are able to minimize the number of health states within the model, making it easier to describe the model structure to the reader [2, 3]. However, individual simulation models do not allow for interaction between the individuals being modeled. For HIV, this is a potential limitation, as the risk of acquiring the infectious disease will be dependent on the number of individuals who already have the infection. In our model we assume HIV incidence varies by sex and age, but not over time across the different strategies examined.

**Appendix B: Model parameter synthesis**

In parameterizing the model we developed a framework for synthesizing the evidence. Our aim was to first use data from the HIVST cluster-randomised trial, or from health economics studies undertaken amongst participants of the trial, where possible. In scenarios where the data was not available, we next sought out findings from published studies undertaken in Malawi, and finally from the wider published literature. When searching the wider published literature, we undertook targeted literature searches in *Pubmed*. The data obtained for the different parameters were all converted to monthly transition probabilities using standard approaches [4].

A targeted literature search provided an efficient approach to obtaining the relevant data [5]. The search involved using MESH terms relating to the parameter of interest and then limiting publications to the Africa region. One researcher scanned the reference list and identified the potentially relevant publications. The full-test of the article was read to determine whether the article provided the relevant data. Where there was more than one data source found, we used the inverse variance weight approach (fixed-effects meta-analysis) to pool the data to estimate a weighted mean and standard error of the weighted mean for parameter inputs.

**Initial characteristics of individuals modeled.**

As part of the cluster-randomised trial, a post-intervention survey was undertaken amongst a representative sample of the intervention and control clusters. This post-intervention study involved collecting data from trial residents, including socio-demographics, HIV testing history, HIV status and CD4 counts. For the majority of these parameters we used the findings in the control clusters.

Table B1 shows the age and sex distribution observed in the control clusters, and the data used in the model. Table B2 shows the HIV prevalence by age and sex observed amongst residents in the control clusters in the post-intervention trial survey. Table B3 shows the CD4 counts observed amongst those who were HIV positive and not on ART in the post-intervention study. This data was used to determine whether individuals had a: CD4 count >500 cells/μl; CD4 count 351-500 cells/μl; CD4 count 201-350 cells/μl; CD4 count 51-200 cells/μl; or CD4 count =< 50 cells/μl; on entry into the model. The CD4 count assigned to individuals on entry into the model was randomly chosen between these ranges.

**Table B1: Sex and age distribution of individuals modeled**

|  | **Mean** | **Distribution** |
| --- | --- | --- |
| Sex: Male (%)  Female (%) | 42%  58% | Uniform distribution |
| Age | 30.02 | Triangular distribution (minimum: 16 years; mean: 30.02 years, max: 92 years) |

Data source: unpublished trial data

**Table B2: HIV prevalence amongst individuals modeled**

| **Age group** | **Male** | | | **Female** | | |
| --- | --- | --- | --- | --- | --- | --- |
|  | **HIV prevalence** | **Parameters for beta distribution** | | **HIV prevalence** | **Parameters for beta distribution** | |
|  |  | **Alpha** | **Beta** |  | **Alpha** | **Beta** |
| 16-19 years | 2.27% | 7 | 301 | 3.75% | 13 | 334 |
| 20-29 years | 3.34% | 14 | 405 | 10.21% | 69 | 607 |
| 30-39 years | 17.44% | 45 | 213 | 27.08% | 114 | 307 |
| 40-49 years | 13.79% | 4 | 25 | 28.57% | 8 | 20 |
| 50+ years | 17.92% | 38 | 174 | 25.00% | 66 | 198 |

Data source: unpublished trial data

**Table B3: CD4 count distribution amongst HIV-positive individuals not on ART**

| **CD4 count amongst control cluster residents** | **N (%)** | **Distribution** |
| --- | --- | --- |
| CD4 count >500 cells/μl | 53 (36.6%) | Dirichlet (53; 34; 33; 22; 3) |
| CD4 count 351-500 cells/μL | 34 (23.4%) |  |
| CD4 count 201-350 cells/μl | 33 (22.8%) |  |
| CD4 count 51-200 cells/μl | 22 (15.2%) |  |
| CD4 count =<50 cells/μl | 3 (2.1%) |  |
| **Total sampled** | 145 |  |

Data source: unpublished trial data

**Uptake of HIV testing and Linkage**

For the HIVST strategies in the model, the likelihood of accessing HIVST was based on findings observed in the cluster-randomised trial [6]. For the model we derived monthly probabilities dependent on the age and sex of individuals modeled. Table B4 shows the monthly probabilities of accessing HIVST used in the model.

For all the strategies in the model, we assumed individuals might continue to access facility-based HTC. To model the likelihood of accessing facility-based HTC we used observed rates in the control clusters of the post-intervention survey undertaken in cluster-randomized trial. For the model we derived monthly probabilities dependent on the age and sex of individuals modeled. Table B5 shows the monthly probabilities of accessing facility-based HTC used in the model.

Individuals who are test HIV-positive may or may not subsequently link into HIV care and treatment services. A prospective cohort study was undertaken in the two main health facilities serving the trial population before the introduction of HIVST [7]. The study estimated the proportion of facility-based HIV testers linking into HIV care, and consequently this data was used to estimate the probability of linkage after facility-based HTC. Of note a systematic review was recently undertaken that estimated linkage into HIV care after facility-based HTC in sub-Saharan Africa [8]. In the review, they estimated approximately 59% (range 35% to 88%) of facility-based HIV testers linked to the HIV clinic for assessment for ART eligibility [8], comparable to the estimate from the study in Malawi [7].

The probability of linking into HIV care after HIVST was based on observed data from the cluster-randomised trial [9]. In the study, the first estimate (524 of the 930 sampled individuals linked into HIV care) excluded those who were already on anti-retroviral therapy at the time of HIVST. The second estimate (524 of the 1257 sampled individuals linked into HIV care) included those already on ART. As the likelihood of linkage into HIV treatment services after HIVST is unlikely to be higher than that after facility-based HTC, we used the second estimate in the analysis. Table B6 shows the data used in the model to determine likelihood of linking into HIV care and treatment services after either facility-based HTC or HIVST, including data used in the sensitivity analysis to examine impact of lower linkage rates after HIVST.

**Table B4: Likelihood of accessing HIVST by age and sex**

| **Age** | **Uptake of HIV self-testing** | | | |
| --- | --- | --- | --- | --- |
|  |  |  |  |  |
|  | **Observed one year probability of accessing HIVST** | | **One month probability of accessing HIVST** | |
|  | **Male** | **Female** | **Male** | **Female** |
| 16-19 years | 0.893 | 0.999 | 0.16993 | 0.61688 |
| 20-29 years | 0.796 | 0.950 | 0.12407 | 0.22092 |
| 30-39 years | 0.600 | 0.779 | 0.07352 | 0.11821 |
| 40-49 years | 0.445 | 0.735 | 0.04788 | 0.10476 |
| 50+ years | 0.408 | 0.582 | 0.04275 | 0.07011 |

Data source: published trial data [6]

**Table B5: Likelihood of accessing facility-based HTC by age and sex**

| **Age** | **Uptake of facility-based HTC** | | | |
| --- | --- | --- | --- | --- |
|  |  |  |  |  |
|  | **Observed one year probability of having accessed facility-based HTC** | | **One month probability of accessing facility-based HTC** | |
|  | **Male** | **Female** | **Male (1)** | **Female (2)** |
| 16-19 years | 0.203 | 0.323 | 0.01872 | 0.03195 |
| 20-29 years | 0.314 | 0.530 | 0.03087 | 0.06091 |
| 30-39 years | 0.261 | 0.444 | 0.02486 | 0.04780 |
| 40-49 years | 0.147 | 0.345 | 0.01317 | 0.03462 |
| 50+ years | 0.249 | 0.265 | 0.02358 | 0.02534 |

Data source: unpublished trial data

**Table B6: Likelihood of linking into HIV care services after HIV testing**

|  | **Observed numbers linked into HIV services** | **Beta distribution** | |
| --- | --- | --- | --- |
|  |  | **Alpha** | **Beta** |
| Linked into HIV care after facility-based HTC | 142 out of 280 | 142 | 138 |
| Linked to HIV care after HIVST | 524 out of 1257 | 524 | 733 |

Published data source [6, 7]

**HIV incidence**

The model was a static model and therefore does not take into account the changing risk of HIV-negative individuals acquiring HIV infection through increased population ART coverage [10]. We undertook a targeted search of the literature to identify studies that have estimated the HIV incidence in African populations since the introduction of ART. We identified one appropriate study that had been undertaken amongst the general population that examined risk of HIV infection during a period of comparable ART coverage to Malawi [11]. The extracted data used to populate the mode is shown in Table B7.

**Table B7: HIV incidence by sex and age**

| **Age group** | **Incidence (per 100 person years)** | | **One month probability** | |
| --- | --- | --- | --- | --- |
|  | **Male** | **Female** | **Male** | **Female** |
| 16-20 years | 0.74 | 4.43 | 0.000616477 | 0.003684861 |
| 20-24 years | 2.53 | 6.49 | 0.002106112 | 0.005393735 |
| 25-29 years | 4.43 | 5.51 | 0.003684861 | 0.004581141 |
| 30-34 years | 2.91 | 3.52 | 0.002422062 | 0.002929035 |
| 35-39 years | 3.16 | 2.26 | 0.002629869 | 0.001881561 |
| 40-44 years | 1.71 | 2.23 | 0.001423985 | 0.001856608 |
| >=45 years | 0.95 | 0.66 | 0.000791353 | 0.000549849 |

Published data source[11]

**HIV and non-HIV associated mortality**

In the model we assumed individuals would be at risk of dying from HIV and non-HIV-associated causes. For non-HIV-associated mortality, we used Malawi specific mortality available through the WHO Global Health Observatory data repository [12]. The data used to model the risk of death from non-HIV-associated causes amongst all individuals are shown in Table B8. As the data was only available as point estimates we did not apply a distribution to represent uncertainty in the PSA.

Targeted literatures search revealed four studies that investigated mortality amongst HIV-positive individuals before starting ART by their CD4 count [13-16]. Only one study provided detailed mortality rates disaggregated by the multiple CD4 count strata used in the model [13]. The data used to model the risk of death amongst HIV-positive individuals not on ART are shown in Table B9.

Since the population scale-up of ART in the region, large cohorts of individuals in different countries in sub-Saharan Africa have been followed up as part of the International Epidemiologic Databases to Evaluate AIDS (IeDEA) collaboration (formerly called ART-Linc) [17]. The collaboration has published several publications on the outcomes of HIV-positive individuals on ART. The publications may use the same cohorts, with longer follow-up, to provide an updated understanding of outcomes for ART patients. Their database of publications relating to Southern Africa was reviewed to obtain the most recent publication (relating to the most recent data), with the longest follow-up and with data on mortality disaggregated by CD4 count.

The data obtained to estimate the probability of death by CD4 count was obtained from one of the studies undertaken using multiple cohorts in South Africa [18]. The study excluded pregnant women and provided data on the risks on mortality in the first three years after starting ART disaggregated by the current CD4 count [18]. The data used to model the risk of death amongst HIV-positive individuals on ART are shown in Table B10.

**Table B8: Non-HIV associated mortality for Malawi**

| **Age group** | **5 year probability (probability of dying between age range)** | | **1 month probability** | |
| --- | --- | --- | --- | --- |
|  | **Male** | **Female** | **Male** | **Female** |
| 15-19 years | 0.012 | 0.010 | 0.000201189 | 0.000167492 |
| 20-24 years | 0.015 | 0.014 | 0.000251862 | 0.000234954 |
| 25-29 years | 0.018 | 0.020 | 0.000302687 | 0.000336655 |
| 30-34 years | 0.027 | 0.030 | 0.000456083 | 0.000507525 |
| 35-39 years | 0.048 | 0.049 | 0.000819501 | 0.000837003 |
| 40-44 years | 0.072 | 0.061 | 0.001244617 | 0.001048447 |
| 45-49 years | 0.091 | 0.066 | 0.001588906 | 0.001137333 |
| 50-54 years | 0.101 | 0.068 | 0.001772964 | 0.001173019 |
| 55-59 years | 0.104 | 0.070 | 0.001828574 | 0.00120878 |
| 60-64 years | 0.122 | 0.089 | 0.002166129 | 0.001552334 |
| 65-69 years | 0.164 | 0.130 | 0.002980992 | 0.002318343 |
| 70-74 years | 0.243 | 0.207 | 0.00462912 | 0.003858073 |
| 75-79 years | 0.356 | 0.317 | 0.007307446 | 0.006334194 |
| 80-84 years | 0.486 | 0.455 | 0.011030909 | 0.010065162 |
| 85-89 years | 0.612 | 0.582 | 0.015655327 | 0.014432732 |
| 90-94 years | 0.709 | 0.685 | 0.020363669 | 0.019068888 |
| 95-99 years | 0.773 | 0.757 | 0.024410545 | 0.023302436 |

Data source: WHO Global Health Observatory data repository [12]

**Table B9: HIV-associated mortality for HIV-positive individuals not on ART**

| **CD count not on ART** | **Observed rate 100 person years (95%CI)** | **One month probability** | | | **Beta distribution** | |
| --- | --- | --- | --- | --- | --- | --- |
|  |  | **Mean** | **Lower 95% CI** | **Upper 95% CI** | **Alpha** | **Beta** |
| CD4 >=500 | 0.6 (0.1, 2.0) | 0.000499875 | 8.33299E-05 | 0.001665279 | 1.533032935 | 3065.299417 |
| CD4 350-499 | 1.6 (0.8, 3.0) | 0.001332445 | 0.000666444 | 0.002496878 | 8.130413984 | 6093.746185 |
| CD4 200-350 | 4.2 (2.8, 5.7) | 0.003493882 | 0.002330613 | 0.004738737 | 32.23027905 | 9192.545418 |
| CD4 100-200 | 17.2 (13.2, 21.2) | 0.0142311 | 0.010939721 | 0.017511526 | 71.01790591 | 4919.313495 |
| CD4 50-99 | 32.4 (23.3, 41.5) | 0.026638758 | 0.019229377 | 0.033992164 | 48.67431166 | 1778.524644 |
| CD4 <50 | 69.5 (55.7, 83.2) | 0.056271412 | 0.045355889 | 0.066984377 | 98.10553729 | 1645.329258 |

Published data source [13]

**Table B10: HIV-associated mortality for HIV-positive individuals on ART**

| **CD4 count on ART** | **Observed rate 100 person years (95%CI)** | **One month probability** | | | **Beta distribution** | |
| --- | --- | --- | --- | --- | --- | --- |
|  |  | **Mean** | **Lower 95% CI** | **Upper 95% CI** | **Alpha** | **Beta** |
| CD4 >350 | 1.4 (1.2, 1.6) | 0.00117 | 0.00100 | 0.00133 | 188.2372536 | 161252.1171 |
| CD4 201-350 | 2.5 (2.3, 2.8) | 0.00208 | 0.00191 | 0.00233 | 384.1900669 | 184219.2038 |
| CD4 101-200 | 5.0 (4.7, 5.4) | 0.00416 | 0.00391 | 0.00449 | 784.0622902 | 187783.1907 |
| CD4 <100 | 14.0 (14.0, 15.0) | 0.01160 | 0.01160 | 0.01242 | 3014.347708 | 256868.4175 |

Published data source [18]

**Risk of HIV associated co-morbidities**

For the risks of co-morbidities two studies were found in the literature [13, 19]. One study provided the risks of HIV associated co-morbidities by CD4 counts for all illnesses concerned except pneumocystis jirovecii pneumonia [13]. For the risk of pneumocystis jirovecii pneumonia we used the data reported in the other study [19]. In the model, we only consider the costs and consequences of hospitalization from these illnesses. We therefore multiplied the risk of suffering these HIV associated co-morbidities by the reported risk of hospitalization from these illness reported in the same study [13].

The model also simulates HIV disease progression to WHO clinical stages 3 or 4 amongst HIV-positive individuals with CD4 counts above 350 cells/μl. This is necessary to take into account that under the 2014 Malawi National guidelines for ART initiation, those whose HIV disease had progressed to WHO clinical stages 3 or 4 were eligible for ART irrespective of their CD4 count. The risk of progression to WHO stage 3 or 4 amongst HIV-positive individuals with CD4 counts above 350 cells/μl was obtained from the same study [13]. The parameters used in the model to estimate the risk of HIV associated co-morbidities and overall likelihood of progression to WHO stage 3 or 4 estimated from this study is shown in Table B11. We did not apply distributions (for the PSA) for the risk of these events.

A literature search did not reveal any studies that provided the risks of severe HIV associated illnesses by CD4 counts for those started on ART. However, a study in South Africa compared the risk of hospitalisations amongst those on ART and those not on ART [20]. The study provided odds ratios for the risk of hospitalisations amongst HIV positive patients on ART relative to HIV positive patients not receiving ART. We therefore multiplied the derived probability from the odds ratios, by the associated risks estimated for those not on ART to determine likelihood of being admitted to hospital for the nine severe HIV-associated illnesses in the model. To model the risk of HIV-associated co-morbidities amongst those on ART we multiplied the probabilities in Table B11 by the probabilities in Table B12.

**Table B11: Risk of Severe HIV associated illness and HIV disease progression by current CD4 count**

|  | **Monthly probability by current CD4 count** | | | | | |
| --- | --- | --- | --- | --- | --- | --- |
|  | **>500 cells/μl** | **350-500 cells/μl** | **200-349 cells/μl** | **100-199 cells/μl** | **50-99 cells/μl** | **<50 cells/μl** |
| Acute diarrhoea | 0.00033 | 0.00011 | 0.00054 | 0.00101 | 0.00099 | 0.00183 |
| Chronic diarrhoea | 0.00000 | 0.00003 | 0.00007 | 0.00034 | 0.00099 | 0.00183 |
| Oesophageal candidiasis | 0.00002 | 0.00003 | 0.00007 | 0.00032 | 0.00103 | 0.00174 |
| Invasive bacterial diseases | 0.00200 | 0.00177 | 0.00518 | 0.00969 | 0.01070 | 0.00958 |
| Pulmonary TB | 0.00032 | 0.00027 | 0.00107 | 0.00257 | 0.00395 | 0.00134 |
| Extra-pulmonary TB | 0.00016 | 0.00016 | 0.00102 | 0.00086 | 0.00155 | 0.00086 |
| Malaria | 0.00092 | 0.00142 | 0.00191 | 0.00258 | 0.00300 | 0.00399 |
| Malignancy (KS/Lymphoma) | 0.00000 | 0.00005 | 0.00009 | 0.00007 | 0.00032 | 0.00054 |
| Pneumocystis Jivorecii pneumonia | 0.00000 | 0.00000 | 0.00025 | 0.00050 | 0.00050 | 0.00673 |
| Cryptococcal meningitis | 0.00000 | 0.00000 | 0.00033 | 0.00042 | 0.00183 | 0.00540 |
| Progression to WHO stage 3 | 0.00615 | 0.00664 |  | | | |
| Progression to WHO stage 4 | 0.00067 | 0.00125 |  |  |  |  |

Published data source[13, 19]

**Table B12: Relative likelihood of HIV-associated illnesses amongst those on ART in comparison to those not on ART**

|  | **Observed odds ratio (95%CI)** | **Probability multiplier** | | | **Beta distribution** | |
| --- | --- | --- | --- | --- | --- | --- |
|  |  | **Mean** | **Lower 95% CI** | **Upper 95% CI** | **Alpha** | **Beta** |
| Multiplier for risk of HIV-associated illness on ART | 0.29 (0.23, 0.36) | 0.22481 | 0.18699 | 0.26471 | 99.45343 | 342.94286 |

Published data source[20]

**Changes in CD4 counts amongst HIV-positive individuals**

The evidence in the literature suggests that the CD4 count falls rapidly after HIV seroconversion and then decreases linearly over time, and increases after initiation of anti-retroviral treatment [21].

We found seven studies describing changes in CD4 counts in HIV-positive individuals living in Africa who had not started ART. One study only provided a description of modeling approaches to estimate changes in CD4 counts [21] and two studies provided mean changes in CD4 counts for the entire sample not disaggregated by CD4 count strata [22, 23]. Consequently the data from these three studies were not utilized. One study investigated the time from HIV infection to the CD4 count falling below 500 cells/μl [24] and four studies provided relevant information on changes in CD4 counts [19, 25-27]. The data provided in these studies were not provided in such a way they could be pooled. We therefore used the mean value, and used a triangular distribution for the PSA with the lowest, mean and highest values reported to characterize the distribution. The data used to model the fall in CD4 count amongst HIV-positive individuals not on ART are shown in Table B13. The data used to determine likelihood of an HIV positive individuals’ CD4 count falling below 500 cells/ul after seroconversion is shown in Table B14.

We found one study that estimated the increase in CD4 count whilst on ART.[18] We used the reported values and assigned a normal distribution, as was used in the analyses undertaken in the study. The study reported weekly increases in CD4 count, which in the model is extrapolated to monthly increases. The data used to model the rise in CD4 count amongst HIV-positive individuals on ART are shown in Table B15.

**Table B13: Fall in CD4 count amongst HIV-positive individuals not on ART**

|  | **Mean monthly decline in CD4 count** | | | |
| --- | --- | --- | --- | --- |
|  | Mean | min | max |  |
| CD4 350-500 | 5.681 | 2.270 | 10.500 | Triangular distribution (min,mean,max) |
| CD4 200-350 | 4.018 | 1.430 | 5.200 |  |
| CD4 50-199 | 4.500 | 3.917 | 5.083 |  |

Published data source [19, 25-27]

**Table B14: Progression to CD4 count below 500 cells/ul after HIV serconversion**

|  | **Observed one-year probability (95%CI)** | **One month probability** | | | **Beta distribution** | |
| --- | --- | --- | --- | --- | --- | --- |
|  |  | **Mean** | **Lower 95% CI** | **Upper 95% CI** | **Alpha** | **Beta** |
| Probability of CD4 count falling to 500 cells/ul after HIV seroconversion | 0.48 (0.47, 0.49) | 0.05304 | 0.05213 | 0.05472 | 6094.481 | 108818.35 |

Published data source [24]

**Table B15: Rise in CD4 count amongst HIV-positive individuals on ART**

|  | **Mean monthly Increase in CD4 count** | | |
| --- | --- | --- | --- |
|  | **Mean** | **Standard error** | **Distribution** |
| Weekly increase in CD4 count on ART | 1.300 | 0.102 | Normal distribution (mean, standard error) |

Published data source [18]

**Outcomes of HIV care and treatment**

Under the 2014 Malawian ART initiation guidelines only those whose CD4 count was below 350 cells/μl, or whose HIV disease had progressed to WHO stage 3 or 4 were eligible to start treatment. Therefore, those who were not eligible under these strategies enter pre-ART care and are asked to return for repeat assessment for ART in 6 months. We undertook a targeted literature search to identify studies that had investigated retention in care amongst those who are not eligible for ART. In the literature a systematic review of this topic had recently been published [28]. We reviewed the papers in the review [27, 29-33], excluding those undertaken amongst pregnant women. Two additional papers, not included in the review, were found that also investigated the likelihood of individuals in pre-ART care returning for repeat assessment for ART eligibility [16, 34]. Table B16 shows the findings from pooling the data from the nine studies using fixed effects meta-analysis.

HIV-positive may not return to continue with their ART. These individuals may have died or defaulted treatment. We identified a recent systematic review that examined retention in care amongst those initiated onto ART [35]. The review highlights that the higher rates of loss occurring during the early stages after starting treatment [35]. The study provides pooled estimates of the proportion of individuals who started ART that are retained in care at 6 month, 12 months, 24 months and thereafter. As those who are not retained in care are either lost to follow-up or died, we used the estimates from the systematic review to determine monthly probabilities of either dying or being lost to care. We then assumed both of these groups of individuals, those retained in care and those not retained in care, would be at risk of HIV-associated mortality (Table B10). Therefore in the model, the monthly probability of being retained on ART, is derived by subtracting the derived monthly probability of loss to follow-up or death from 1. Table B17 shows the parameters used in the model to determine retention on ART after starting treatment by the month since starting ART.

**Table B16: Likelihood of returning for repeat assessment for ART initiation**

|  | **Pooled estimate** | | | **Beta distribution** | |
| --- | --- | --- | --- | --- | --- |
|  | **Mean** | **Lower 95% CI** | **Upper 95% CI** | **Alpha** | **Beta** |
| Probability of returning for repeat assessment for ART initiation | 0.571 | 0.560 | 0.580 | 9719.824497 | 7293.85384 |

Published data source [27, 29-33]

**Table B17: Retention on ART by month since starting treatment**

|  | **Observed ART retention rates** | **Monthly probability of lost to follow-up or death during time period** | | | **Beta Distribution** | |
| --- | --- | --- | --- | --- | --- | --- |
|  | **Mean %**  **(95% CI)** | **Mean** | **Lower 95% CI** | **Upper 95% CI** | **Alpha** | **Beta** |
| Retained in ART care: 0-6 month since starting ART | 86.1 (84.6, 87.4) | 0.0246 | 0.0275 | 0.0222 | 324.7642 | 12858.3055 |
| Retained in ART care 7-12 month since starting ART | 80.2 (78.0, 82.4) | 0.0118 | 0.0134 | 0.0098 | 155.4312 | 13060.0765 |
| Retained in ART care 13-24 month since starting ART | 76.1 (72.4, 79.7) | 0.0044 | 0.0062 | 0.0028 | 24.9457 | 5692.10088 |
| Retained in ART care 25+ month since starting ART | 72.3 (67.4, 76.9) | 0.0043 | 0.0059 | 0.0030 | 31.4726 | 7357.18486 |

Published data source [35]

**Health provider and societal costs in 2014 International dollars**

Table B17 shows the health provider costs and societal costs estimated in 2014 international dollars. These estimates were derived from the three primary health economics studies undertaken in the trial population [36] and in Queen Elizabeth Central Hospital [37].

**Table B18: Health provider and societal costs for model (2014 International Dollars)**

| Cost Parameter | | 2014 INT Dollars | | | | | | | Distribution |  |
| --- | --- | --- | --- | --- | --- | --- | --- | --- | --- | --- |
|  |  | **Health provider costs** | | |  | **Societal costs** | | |  |  |
|  |  | Base case | Low | High |  | Base case | Low | High |  | |
| Facility-based HTC episode | | 20.44 | 20.25 | 25.18 |  | 26.78 | 24.94 | 28.63 |  | |
| HIV self-testing episode | | 17.25 | 14.25 | 22.42 |  | 17.62 | 15.09 | 20.16 |  | |
| Assessment for ART eligibility for all clients | | 59.83 | 57.29 | 62.36 |  | 68.70 | 65.10 | 72.29 |  | |
| Annual cost of ART for facility HTC clients | | 229.85 | 218.19 | 241.50 |  | 268.45 | 246.75 | 290.15 |  | |
| Annual cost of ART for facility HIVST clients | | 217.04 | 198.37 | 235.71 |  | 259.86 | 222.42 | 297.31 | Gamma | |
| Cost of hospital admission for severe HIV associated illness | Acute diarrhoea | 803.60 | 341.95 | 1265.25 |  | 1305.25 | 440.98 | 2169.53 |  | |
|  | Chronic diarrhoea | 609.07 | 247.00 | 971.13 |  | 686.42 | 304.20 | 1068.64 |  | |
|  | Oesophageal candidiasis | 395.98 | 160.40 | 631.56 |  | 467.81 | 141.63 | 794.00 |  |  |
|  | Invasive bacterial diseases | 581.11 | 517.91 | 644.31 |  | 683.21 | 597.16 | 769.26 |  | |
|  | Pulmonary tuberculosis | 1147.37 | 889.83 | 1404.92 |  | 1540.76 | 1152.12 | 1929.39 |  | |
|  | Extra-pulmonary tuberculosis | 1294.81 | 1024.16 | 1565.46 |  | 2060.80 | 1399.39 | 2722.21 |  | |
|  | Malaria | 488.44 | 246.56 | 730.32 |  | 929.60 | 105.93 | 1753.27 |  | |
|  | Malignancy (KS/Lymphoma) | 637.17 | 507.12 | 767.23 |  | 843.02 | 634.03 | 1052.01 |  | |
|  | Pneumocystis Jivorecii pneumonia | 849.35 | 702.89 | 995.81 |  | 1042.65 | 765.35 | 1319.94 |  | |
|  | Cryptococcal meningitis | 1583.26 | 1304.33 | 1862.20 |  | 1948.54 | 1539.54 | 2357.55 |  | |

HTC: HIV testing and counselling ART: Anti-retroviral treatment

HIVST: HIV self testing KS: Kaposi’s sarcoma

**Appendix C: Model validation**

**Overview**

The model was validated using two broad approaches. Firstly face validity of the model was undertaken by reviewing the literature around economic evaluation of HIV interventions to determine best practices and approaches to modeling HIV interventions. Discussion with experts in the field of HIV were undertaken to determine appropriate clinical pathways for HIV disease progression and outcomes of undergoing HIV testing and HIV treatment.

Secondly, we undertook model verification and examined the external validity of the model [38]. We examined outputs from the model to findings in the HIVST cluster-randomized trial, findings from published studies undertaken in Malawi and sub-Saharan Africa. We compared the model outputs from the two scenarios where ART initiation followed the 2010 WHO ART guidelines as these were being followed at the time of the trial and in the other studies.

**Findings from model validation**

We evaluated the model predicted uptake of both facility HTC and HIVST, and the HIV prevalence amongst HIV testers to findings in the HIVST cluster-randomized trial and published findings from the region.

We modelled the uptake of facility HTC and HIVST to vary by the age and sex of individuals simulated. The model tracks the proportion of the total population modelled who accessed either modality of HIV testing every year. Table C1 shows the outputs from the model simulations and compares to findings observed in Malawi and in the HIVST trial. The proportion of the total population who accessed facility-based HTC every year in the first three years ranged from 24.4% to 32.7%, and comparable to that observed in a separate study undertaken in Blantyre, Malawi prior to implementing HIV self-testing (22.6%) [39]. In the model simulations, the proportion of the total population who accessed HIVST every year in the first three years ranged from 37.6% to 61.4%. This was lower than that found in the HIVST cluster randomised trial (74.4% to 76.5%). This difference may in part be explained by the fact that in the cluster randomised trial the population may migrate in and out of the study clusters and consequently the denominator used to estimate population uptake HIVST in the trial may be an underestimate [6].

The model tracks the proportion of facility HTC and HIV self-testing clients who test HIV-positive. In the model simulations, the HIV prevalence amongst facility HTC clients was comparable to that observed in another study in Malawi (Table C1) [40]. In the model simulations, the HIV prevalence amongst HIV self-testers was not too dissimilar to that observed in the HIVST cluster-randomised trial.

**Table C1: Model validation of uptake of facility HIV testing or HIV self-testing**

|  | **Model findings** | | **Real world data** | |
| --- | --- | --- | --- | --- |
|  | **Model strategy** | **Simulated finding** | **Observed finding** | **Source** |
| Annual population uptake of facility HTC | Facility HTC  2010 WHO ART | Year 1: 32.7%  Year 2: 25.9%  Year 3: 22.5% | 22.6% | [39] |
| Annual population uptake of HIVST | Facility HTC + HIVST  2010 WHO ART | Year 1: 61.5%  Year 2: 42.5%  Year 3: 38.4% | Year 1: 76.5%  Year 2: 74.4% | [6] |
| HIV prevalence amongst facility HTC clients | Facility HTC  2010 WHO ART | Year 1: 19.8%  Year 2: 19.8%  Year 3: 19.3% | 17.7% to 19.2% | [40] |
| HIV prevalence amongst HIV self-testers | Facility HTC + HIVST  2010 WHO ART | Year 1: 14.8%  Year 2: 12.1%  Year 3: 11.5% | Year 1: 10.1%-11.8%  Year 2: 6.8%-7.3% | [6] |

We compared the model outputs pertaining to linkage into HIV treatment and outcomes of those on HIV treatment to previous studies to ensure the calculations being undertaken in the model reflect real world findings. In the model we tracked the proportion of individuals who tested HIV-positive through either modality of HIV testing who subsequently linked into HIV treatment services and who initiated ART. The linkage rate into HIV treatment services after facility HTC and HIVST observed in the model simulations was comparable to the parameter used to model linkage (Table C2).

**Table C2: Model validation of linkage into HIV treatment after HIV testing**

|  | **Model findings** | | **Real world data** | |
| --- | --- | --- | --- | --- |
|  | **Model strategy** | **Simulated finding** | **Observed finding** | **Source** |
| Proportion of HIV-positive facility HTC clients attending HIV clinic | Facility HTC  2010 WHO ART | Year 1: 51.1%  Year 2: 55.3%  Year 3: 55.3% | 50.7% | [7] |
| Proportion of HIV-positive HIVST clients attending HIV clinic | Facility HTC + HIVST  2010 WHO ART | Year 1: 41.9%  Year 2: 41.9%  Year 3: 41.7% | 56.3% | [6] |
| Proportion of HIV-positive facility HTC clients starting ART | Facility HTC  2010 WHO ART | Year 1: 26.2%  Year 2: 26.2%  Year 3: 24.9% | 31.1% | [7] |
| Proportion of HIV-positive HIVST clients starting ART | Facility HTC + HIVST  2010 WHO ART | Year 1: 22.6%  Year 2: 19.2%  Year 3: 17.2% | *27.6% | [6] |

*Estimated: 41.7% HIV self-testers attended clinic, with 66.3% having CD4 count<350 cells/μl (would of started ART) [6]

In the model we simulate HIV disease progression, response to ART and mortality dependent on the CD4 count of individuals simulated. We undertook model validation checks on this component of the model by first comparing the CD4 count on HIV testing and on ART initiation, and also by comparing mortality on ART from the model simulations to findings observed in the real world.

Table C3 shows the model validation checks on the CD4 count on accessing HIV testing and on initiation of antiretroviral therapy simulated by the model and that observed in the real world. The CD4 count on accessing facility HTC in the model simulations was marginally higher than that observed in other studies. We are not aware of any studies that have measured CD4 count at the time of HIV self-testing.

In the model simulation we found that the CD4 count of facility HIV testers and HIV self-testers that start ART to be comparable to that observed in Blantyre, and in the HIVST cluster randomised trial.

**Table C3: Model validation of CD4 count amongst HIV testers and ART initiators**

|  | **Model findings** | | **Real world data** | |
| --- | --- | --- | --- | --- |
|  | **Model strategy** | **Simulated finding** | **Observed finding** | **Source** |
| CD4 count amongst facility HTC clients at HIV testing | Facility HTC  2010 WHO ART | Year 1: 366 cells/μl  Year 2: 365 cells/μl  Year 3: 362 cells/μl | Median: 300 cells/μl | [41] |
| CD4 count amongst HIVST clients at HIV testing | Facility HTC + HIVST  2010 WHO ART | Year 1: 368 cells/μl  Year 2: 374 cells/μl  Year 3: 378 cells/μl | Not available |  |
| CD4 count amongst facility HTC clients at ART initiation | Facility HTC  2010 WHO ART | Year 1: 234 cells/μl  Year 2: 244 cells/μl  Year 3: 247 cells/μl | Median: 240 cells/μl | [7] |
| CD4 count amongst HIVST clients at ART initiation | Facility HTC + HIVST  2010 WHO ART | Year 1: 240 cells/μl  Year 2: 247 cells/μl  Year 3: 256 cells/μl | Median: 250 cells/μl | [6] |

Table C4 shows the model validation checks on the outcomes of receiving ART. For this we compared the mortality in the first year of receiving ART by the CD4 count on ART initiation. We found the first year mortality seen in the model simulations were comparable to that observed in real world studies undertaken in the region.

**Table C4: Model validation of outcomes on Anti-retroviral therapy**

| **CD4 count on ART initiation** | **Mortality at one-year after starting ART** | | | |
| --- | --- | --- | --- | --- |
|  | **Model findings** | | **Real world data** | |
|  | **Model strategy** | **Simulated finding** | **Observed finding** | **Source** |
| >=200 cells/μl | Facility HTC  2010 WHO ART | 5.1% | 5.1% | [42] |
| 100-199 cells/μl | Facility HTC  2010 WHO ART | 6.9% | 5.5% |  |
| 50-99 cells/μl | Facility HTC  2010 WHO ART | 10.5% | 8.7% |  |
| 25-49 cells/μl | Facility HTC  2010 WHO ART | 14.2% | 16.6% |  |
| <25 cells/μl | Facility HTC  2010 WHO ART | 15.2% | 21.4% |  |

**Appendix D: Primary cost-effectiveness analysis in International Dollars**

Table D1 shows the findings from the primary analysis from both the health provider and societal perspective with costs estimated in 2014 International dollars.

**Table D1: Cost-effectiveness findings from primary analysis and 20 year time horizon (2014 INT Dollars)**

| Perspective | HIV testing strategy | ART initiation guideline | Discounted mean costs and QALYs per person | | | | ICER | **Probability cost-effective at cost-effectiveness threshold (INT$ per QALY) | | | |
| --- | --- | --- | --- | --- | --- | --- | --- | --- | --- | --- | --- |
|  |  |  | 2014 INT Dollars | | QALYs | |  |  |  |  |  |
|  |  |  | Mean cost  (*95% CrI) | Incremental cost  (*95% CrI) | Mean Effectiveness  (*95% CrI) | Incremental Effectiveness  (*95% CrI) | 2014 INT$ per QALY  (*95% CrI) | 0 | 250 | 500 | 750 |
| Health Provider | Facility HTC | 2010 WHO ART | 494.62  (458.03, 534.16) | - | 11.64  (11.43, 11.86) | - | - | 1.000 | 0.393 | 0 | 0 |
|  | Facility HTC | 2015 WHO ART | 539.51  (504.49, 577.60) | 44.88  (30.03, 63.02) | 11.82  (11.62, 12.03) | 0.18  (0.12, 0.25) | 244.10  (203.51, 303.11) | 0 | 0.562 | 0.032 | 0.002 |
|  | Facility HTC & HIVST | 2010 WHO ART | 618.40  (578.59, 658.55) | - | 11.99  (11.80, 12.18) | - | ED*** | 0 | 0.007 | 0 | 0 |
|  | Facility HTC & HIVST | 2015 WHO ART | 676.76  (638.86, 716.60) | 137.26  (106.12, 168.49) | 12.23  (12.06, 12.40) | 0.40  (0.28, 0.53) | 339.34  (252.50, 477.95) | 0 | 0.038 | 0.968 | 0.998 |
| Societal | Facility HTC | 2010 WHO ART | 610.97  (558.14, 665.90) | - | 11.64  (11.43, 11.86) | - | - | 1.000 | 0.671 | 0 | 0 |
|  | Facility HTC | 2015 WHO ART | 659.89  (608.61, 713.53) | 48.91  (31.95, 70.16) | 11.82  (11.62, 12.03) | 0.18  (0.12, 0.25) | 267.01  (211.98, 335.45) | 0 | 0.301 | 0.073 | 0.001 |
|  | Facility HTC & HIVST | 2010 WHO ART | 742.06  (687.49, 797.32) | - | 11.99  (11.80, 12.18) | - | ED*** | 0 | 0.008 | 0 | 0 |
|  | Facility HTC & HIVST | 2015 WHO ART | 807.71  (753.40, 862.65) | 147.82  (106.03, 189.58) | 12.23  (12.06, 12.40) | 0.40  (0.28, 0.53) | 365.40  (262.09, 523.94) | 0 | 0.020 | 0.926 | 0.999 |

HTC: HIV testing and counselling HIVST: HIV self-testing ART: Anti-retroviral treatment CrI: Credible interval

QALYS: Quality-adjusted life year ICER: Incremental cost-effectiveness ratio

2010 WHO ART initiation guidelines: CD4 count <350 cells/mm^3^ or WHO stage 3 or 4

2015 WHO ART initiation guidelines: start ART irrespective of CD4 count or WHO stage

*95% CrI: represents the 2.5^th^ and 97.5^th^ percentile from the distribution of results from all the simulations

**Probability represents the proportion of all simulations where the estimated ICER was below the specified cost-effectiveness threshold. Total may not add up to 1.0 as for some simulations, no single scenario was found most cost-effective at given ICER threshold

***Extended dominance: The ICER for this strategy higher than the next more effective strategy.

**Appendix E: Deterministic sensitivity analysis**

Tables E1-E4 shows the findings from the deterministic sensitivity analysis when the analysis was undertaken from the health provider perspective and over a 20 year time horizon. Each table shows the discounted incremental costs, incremental QALYs and ICER. This is shown comparing the strategies that were not excluded due to extended dominance.

Figure E1 shows a tornado plot from the deterministic sensitivity analysis comparing the strategy of facility HTC and using 2015 WHO ART guidelines to the strategy of facility HTC and using 2010 WHO ART guidelines. The tornado diagram comparing the strategy of facility HTC plus HIVST and using 2015 WHO ART guidelines to the strategy of facility HTC and using 2015 WHO ART guidelines is shown in the main text (Figure 3).

**Table E1: Findings from deterministic sensitivity analysis**

|  |  | Discounted incremental costs  (2014 US$) | | Discounted incremental QALYs | | ICER (2014 US$ per QALY) | |
| --- | --- | --- | --- | --- | --- | --- | --- |
|  |  | (Facility HTC & WHO 2015) **V** (Facility HTC + WHO 2010) | (Facility HTC + HIVST & WHO 2015) **V** (Facility HTC & WHO 2015) | (Facility HTC & WHO 2015) **V** (Facility HTC & WHO 2010) | (Facility HTC + HIVST & WHO 2015) **V** (Facility HTC & WHO 2015) | (Facility HTC & WHO 2015) **V** (Facility HTC & WHO 2010) | (Facility HTC + HIVST & WHO 2015) **V** (Facility HTC & WHO 2015) |
| Base case (deterministic) |  | **37.33** | **105.33** | **0.1423** | **0.3482** | **262.36** | **302.46** |
| UV1: HIV prevalence | low | 33.07 | 97.65 | 0.1411 | 0.3508 | 234.34 | 278.40 |
|  | high | 36.86 | 111.83 | 0.1249 | 0.4913 | 295.03 | 227.64 |
| UV2: HIV incidence | low | 20.41 | 91.97 | 0.0703 | 0.3790 | 290.43 | 242.69 |
|  | high | 76.61 | 146.08 | 0.3356 | 0.6185 | 228.30 | 236.19 |
| UV3: Annual uptake of Facility HTC | low | 24.64 | 157.50 | 0.1164 | 0.7167 | 211.70 | 219.75 |
|  | high | 44.65 | 61.34 | 0.2093 | 0.1640 | 213.38 | 374.04 |
| UV4: Annual uptake of HIVST | low | 40.92 | 75.99 | 0.1819 | 0.3352 | 224.98 | 226.73 |
|  | high | 38.54 | 199.80 | 0.1687 | 0.7270 | 228.52 | 274.82 |
| UV5: Linkage after Facility HTC | low | 36.98 | 109.49 | 0.1715 | 0.3963 | 215.67 | 276.28 |
|  | high | 40.81 | 96.65 | 0.1690 | 0.3080 | 241.42 | 313.83 |
| UV6: Linkage after HIVST | low | 35.49 | 100.52 | 0.1338 | 0.3396 | 265.31 | 295.99 |
|  | high | 39.36 | 104.74 | 0.1585 | 0.3430 | 248.28 | 305.35 |
| UV7: HIV mortality: not on ART | low | 34.28 | 106.69 | 0.1077 | 0.4236 | 318.35 | 251.89 |
|  | high | 38.46 | 111.58 | 0.1711 | 0.4133 | 224.83 | 269.97 |
| UV8: HIV mortality: on ART | low | 40.18 | 108.51 | 0.1655 | 0.3795 | 242.78 | 285.96 |
|  | high | 36.04 | 103.60 | 0.1335 | 0.3469 | 270.00 | 298.64 |
| UV9: Mean change in CD4 count: Not on ART | low | 49.27 | 98.34 | 0.1521 | 0.3328 | 323.99 | 295.49 |
|  | high | 36.30 | 91.99 | 0.1844 | 0.3350 | 196.86 | 274.62 |
| UV10: Mean change in CD4 count: on ART | low | 36.54 | 103.12 | 0.1445 | 0.3413 | 252.90 | 302.16 |
|  | high | 37.65 | 104.11 | 0.1395 | 0.3506 | 269.96 | 296.95 |
| UV11: Pre-ART returning for ART assessment | low | 38.08 | 105.33 | 0.1462 | 0.3482 | 260.53 | 302.47 |
|  | high | 36.95 | 105.33 | 0.1391 | 0.3482 | 265.60 | 302.47 |
| UV12: Retention on ART | low | 30.06 | 114.90 | 0.1048 | 0.3281 | 286.91 | 350.25 |
|  | high | 30.09 | 110.41 | 0.0832 | 0.3687 | 361.78 | 299.44 |
| UV13: Cost of Facility HTC | low | 37.41 | 106.84 | 0.1423 | 0.3482 | 262.91 | 306.80 |
|  | high | 37.24 | 103.50 | 0.1423 | 0.3482 | 261.69 | 297.21 |
| UV14: Cost of HIVST | low | 37.33 | 100.98 | 0.1423 | 0.3482 | 262.36 | 289.97 |
|  | high | 37.33 | 112.65 | 0.1423 | 0.3482 | 262.36 | 323.48 |

UV: Univariate/one-way sensitivity analysis

**Table E2: Findings from deterministic sensitivity analysis**

|  |  | Discounted incremental costs  (2014 US$) | | Discounted incremental QALYs | | ICER (2014 US$ per QALY) | |
| --- | --- | --- | --- | --- | --- | --- | --- |
|  |  | (Facility HTC & WHO 2015) **V** (Facility HTC + WHO 2010) | (Facility HTC + HIVST & WHO 2015) **V** (Facility HTC & WHO 2015) | (Facility HTC & WHO 2015) **V** (Facility HTC & WHO 2010) | (Facility HTC + HIVST & WHO 2015) **V** (Facility HTC & WHO 2015) | (Facility HTC & WHO 2015) **V** (Facility HTC & WHO 2010) | (Facility HTC + HIVST & WHO 2015) **V** (Facility HTC & WHO 2015) |
| Base case (deterministic) |  | **37.33** | **105.33** | **0.1423** | **0.3482** | **262.36** | **302.46** |
| UV15: Cost of ART assessment | low | 37.47 | 105.25 | 0.1423 | 0.3482 | 263.36 | 302.23 |
|  | high | 37.17 | 105.43 | 0.1423 | 0.3482 | 261.25 | 302.75 |
| UV16: Annual cost of ART provision after facility HTC | low | 36.18 | 107.52 | 0.1423 | 0.3482 | 254.27 | 308.75 |
|  | high | 38.49 | 103.13 | 0.1423 | 0.3482 | 270.48 | 296.15 |
| UV17: Annual cost of ART provision after HIVST | low | 37.33 | 96.42 | 0.1423 | 0.3482 | 262.36 | 276.88 |
|  | high | 37.33 | 114.21 | 0.1423 | 0.3482 | 262.36 | 327.96 |
| UV18: Cost: Acute diarrhoea | low | 37.50 | 105.70 | 0.1423 | 0.3482 | 263.51 | 303.53 |
|  | high | 37.17 | 104.95 | 0.1423 | 0.3482 | 261.21 | 301.37 |
| UV19: Cost: Chronic diarrhoea | low | 37.45 | 105.46 | 0.1423 | 0.3482 | 263.17 | 302.84 |
|  | high | 37.22 | 105.30 | 0.1423 | 0.3482 | 261.55 | 302.38 |
| UV20: Cost: Oesophageal candidiasis | low | 37.38 | 105.47 | 0.1423 | 0.3482 | 262.70 | 302.87 |
|  | high | 37.28 | 105.19 | 0.1423 | 0.3482 | 262.02 | 302.06 |
| UV21: Cost: Invasive bacterial diseases | low | 37.66 | 105.69 | 0.1423 | 0.3482 | 264.66 | 303.50 |
|  | high | 37.00 | 104.97 | 0.1423 | 0.3482 | 260.07 | 301.43 |
| UV22: Cost: Pulmonary tuberculosis | low | 37.62 | 105.74 | 0.1423 | 0.3482 | 264.39 | 303.64 |
|  | high | 37.04 | 104.93 | 0.1423 | 0.3482 | 260.33 | 301.32 |
| UV23: Cost: Extra-pulmonary tuberculosis | low | 37.51 | 105.58 | 0.1423 | 0.3482 | 263.58 | 303.18 |
|  | high | 37.16 | 105.08 | 0.1423 | 0.3482 | 261.14 | 301.75 |
| UV24: Cost: Malaria | low | 37.63 | 105.86 | 0.1423 | 0.3482 | 264.47 | 303.99 |
|  | high | 37.03 | 104.81 | 0.1423 | 0.3482 | 260.25 | 300.97 |
| UV25: Cost: Malignancy (KS/Lymphoma) | low | 37.34 | 105.36 | 0.1423 | 0.3482 | 262.42 | 302.55 |
|  | high | 37.32 | 105.31 | 0.1423 | 0.3482 | 262.31 | 302.41 |
| UV26: Cost: Pneumocystis Jivorecii pneumonia | low | 37.41 | 105.51 | 0.1423 | 0.3482 | 262.90 | 302.98 |
|  | high | 37.25 | 105.16 | 0.1423 | 0.3482 | 261.82 | 301.98 |
| UV27: Cost: Cryptococcal Meningitis | low | 37.40 | 105.69 | 0.1423 | 0.3482 | 262.82 | 303.50 |
|  | high | 37.27 | 104.97 | 0.1423 | 0.3482 | 261.90 | 301.43 |

UV: Univariate/one-way sensitivity analysis

**Table E3: Findings from deterministic sensitivity analysis**

|  |  | Discounted incremental costs  (2014 US$) | | Discounted incremental QALYs | | ICER (2014 US$ per QALY) | |
| --- | --- | --- | --- | --- | --- | --- | --- |
|  |  | (Facility HTC & WHO 2015) **V** (Facility HTC + WHO 2010) | (Facility HTC + HIVST & WHO 2015) **V** (Facility HTC & WHO 2015) | (Facility HTC & WHO 2015) **V** (Facility HTC & WHO 2010) | (Facility HTC + HIVST & WHO 2015) **V** (Facility HTC & WHO 2015) | (Facility HTC & WHO 2015) **V** (Facility HTC & WHO 2010) | (Facility HTC + HIVST & WHO 2015) **V** (Facility HTC & WHO 2015) |
| Base case (deterministic) |  | **37.33** | **105.33** | **0.1423** | **0.3482** | **262.36** | **302.46** |
| UV28: Utility score: HIV-positive not on ART, CD4 >200 cells/μl | low | 37.33 | 105.33 | 0.1582 | 0.3641 | 235.95 | 289.28 |
|  | high | 37.33 | 105.33 | 0.1244 | 0.3303 | 300.09 | 318.92 |
| UV29: Utility score: HIV-positive not on ART, CD4 51 to 200 cells/μl | low | 37.33 | 105.33 | 0.1444 | 0.3527 | 258.45 | 298.68 |
|  | high | 37.33 | 105.33 | 0.1402 | 0.3438 | 266.33 | 306.34 |
| UV30: Utility score: HIV-positive not on ART, CD4 count <=50 cells/μl | low | 37.33 | 105.33 | 0.1485 | 0.3533 | 251.42 | 298.10 |
|  | high | 37.33 | 105.33 | 0.1459 | 0.3511 | 255.94 | 300.00 |
| UV31: Utility score: Increase over first year on ART for facility-HTC clients | low | 37.33 | 105.33 | 0.1423 | 0.3484 | 262.41 | 302.29 |
|  | high | 37.33 | 105.33 | 0.1422 | 0.3481 | 262.45 | 302.56 |
| UV32: Utility score: Increase over first year on ART for HIVST clients | low | 37.33 | 105.33 | 0.1423 | 0.3475 | 262.36 | 303.12 |
|  | high | 37.33 | 105.33 | 0.1423 | 0.3483 | 262.36 | 302.42 |
| UV33: Utility score: Acute diarrhoea | low | 37.33 | 105.33 | 0.1423 | 0.3483 | 262.33 | 302.43 |
|  | high | 37.33 | 105.33 | 0.1423 | 0.3482 | 262.39 | 302.50 |
| UV34: Utility score: Chronic diarrhoea | low | 37.33 | 105.33 | 0.1423 | 0.3483 | 262.34 | 302.45 |
|  | high | 37.33 | 105.33 | 0.1423 | 0.3482 | 262.38 | 302.47 |
| UV35: Utility score: Oesophageal candidiasis | low | 37.33 | 105.33 | 0.1423 | 0.3483 | 262.34 | 302.44 |
|  | high | 37.33 | 105.33 | 0.1423 | 0.3482 | 262.38 | 302.49 |
| UV36: Utility score: Invasive bacterial diseases | low | 37.33 | 105.33 | 0.1423 | 0.3483 | 262.27 | 302.42 |
|  | high | 37.33 | 105.33 | 0.1422 | 0.3482 | 262.45 | 302.51 |
| UV37: Utility score: Pulmonary tuberculosis | low | 37.33 | 105.33 | 0.1423 | 0.3483 | 262.33 | 302.44 |
|  | high | 37.33 | 105.33 | 0.1423 | 0.3482 | 262.40 | 302.49 |
| UV38: Utility score: Extra-pulmonary tuberculosis | low | 37.33 | 105.33 | 0.1423 | 0.3483 | 262.34 | 302.45 |
|  | high | 37.33 | 105.33 | 0.1423 | 0.3482 | 262.39 | 302.48 |
| UV39: Utility score: Malaria | low | 37.33 | 105.33 | 0.1423 | 0.3483 | 262.28 | 302.40 |
|  | high | 37.33 | 105.33 | 0.1422 | 0.3482 | 262.44 | 302.53 |
| UV40: Utility score: Malignancy (KS/Lymphoma) | low | 37.33 | 105.33 | 0.1423 | 0.3482 | 262.36 | 302.46 |
|  | high | 37.33 | 105.33 | 0.1423 | 0.3482 | 262.36 | 302.47 |
| UV41: Utility score: Pneumocystis Jivorecii pneumonia | low | 37.33 | 105.33 | 0.1423 | 0.3483 | 262.33 | 302.43 |
|  | high | 37.33 | 105.33 | 0.1423 | 0.3482 | 262.39 | 302.50 |

UV: Univariate/one-way sensitivity analysis

**Table E4: Findings from deterministic sensitivity analysis**

|  |  | Discounted incremental costs  (2014 US$) | | Discounted incremental QALYs | | ICER (2014 US$ per QALY) | |
| --- | --- | --- | --- | --- | --- | --- | --- |
|  |  | (Facility HTC & WHO 2015) **V** (Facility HTC + WHO 2010) | (Facility HTC + HIVST & WHO 2015) **V** (Facility HTC & WHO 2015) | (Facility HTC & WHO 2015) **V** (Facility HTC & WHO 2010) | (Facility HTC + HIVST & WHO 2015) **V** (Facility HTC & WHO 2015) | (Facility HTC & WHO 2015) **V** (Facility HTC & WHO 2010) | (Facility HTC + HIVST & WHO 2015) **V** (Facility HTC & WHO 2015) |
| Base case (determinisitic) |  | **37.33** | **105.33** | **0.1423** | **0.3482** | **262.36** | **302.46** |
| UV42: Utility score: Cryptococcal Meningitis | low | 37.33 | 105.33 | 0.1423 | 0.3483 | 262.36 | 302.45 |
|  | high | 37.33 | 105.33 | 0.1423 | 0.3482 | 262.37 | 302.48 |
| UV43: Discount rate (0-6%) | low | 47.51 | 138.76 | 0.0899 | 0.2459 | 528.42 | 564.39 |
|  | high | 30.16 | 82.82 | 0.1809 | 0.4191 | 166.75 | 197.61 |
| MV1: UV3 + UV4 | low | 18.12 | 124.44 | 0.0758 | 0.6668 | 239.04 | 186.62 |
|  | high | 18.13 | 66.64 | 0.0860 | 0.2245 | 210.93 | 296.86 |
| MV2: UV3 + UV4 + UV1 | low | 18.93 | 98.56 | 0.0929 | 0.4872 | 203.82 | 202.30 |
|  | high | 68.08 | 108.33 | 0.3001 | 0.2683 | 226.87 | 403.78 |
| MV3: UV3 + UV4 + UV1 + UV2 | low | 15.20 | 74.52 | 0.0859 | 0.3466 | 176.92 | 215.02 |
|  | high | 102.95 | 149.70 | 0.3863 | 0.4667 | 266.52 | 320.77 |
| MV4: UV3+ UV4 + UV1 + UV2 + UV17 | low | 15.20 | 70.49 | 0.0859 | 0.3466 | 176.92 | 203.41 |
|  | high | 102.95 | 198.03 | 0.3863 | 0.4667 | 266.52 | 424.32 |
| MV5: UV4 + UV1 + UV2 + UV17 | low | 17.53 | 51.81 | 0.0803 | 0.2021 | 218.24 | 256.41 |
|  | high | 73.25 | 156.04 | 0.3118 | 0.7553 | 234.92 | 206.60 |
| MV6: UV4+ UV1 + UV2 + UV17 + UV7 | low | 12.61 | 54.54 | 0.0466 | 0.2265 | 270.28 | 240.84 |
|  | high | 84.75 | 157.91 | 0.4000 | 0.7403 | 211.86 | 213.31 |
| MV7: UV1 + UV2 + UV17 + UV7 + UV5 | low | 17.52 | 75.71 | 0.0767 | 0.2522 | 228.30 | 300.25 |
|  | high | 81.82 | 177.43 | 0.3738 | 0.8255 | 218.90 | 214.95 |
| MV8: UV3 + UV1 + UV2 + UV17 + UV7 + UV5 | low | 6.59 | 110.52 | 0.0330 | 0.5482 | 199.53 | 201.62 |
|  | high | 55.25 | 144.95 | 0.2308 | 0.6122 | 239.36 | 236.76 |

UV: Univariate/one-way sensitivity analysis

MV: Multivariate sensitivity analysis

**Figure E1: Tornado diagram showing findings from deterministic sensitivity analysis**

**
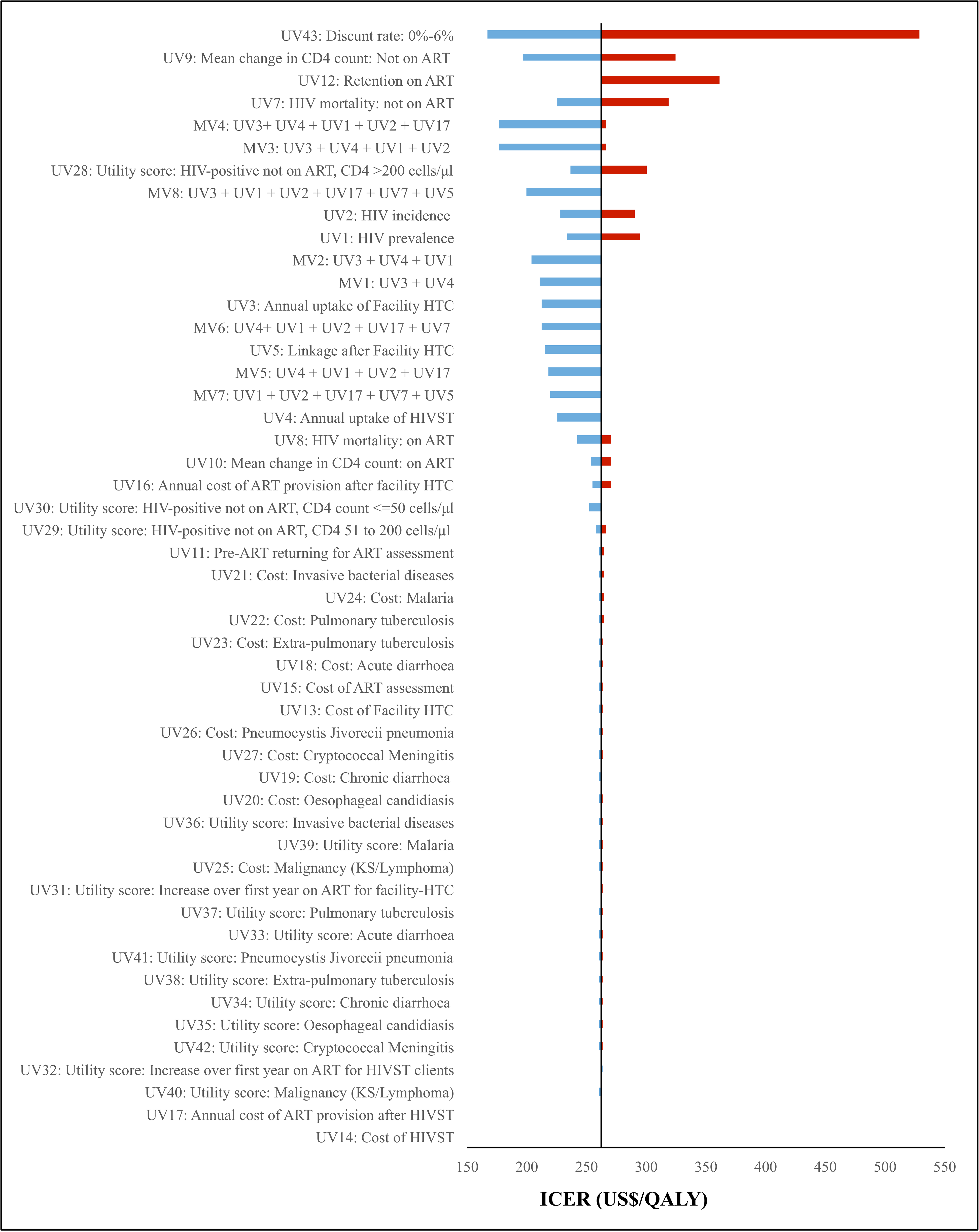
**

UV: Univariate/one-way sensitivity analysis MV: Multivariate sensitivity analysis

ART: Anti-retroviral treatment HTC: HIV testing and counselling

HIVST: HIV self-testing ICER: Incremental cost-effectiveness ratio

QALY: Quality-adjusted life year KS: Kaposi’s sarcoma

**References**

1. O'Hagan A, Stevenson M, Madan J. Monte Carlo probabilistic sensitivity analysis for patient level simulation models: efficient estimation of mean and variance using ANOVA. Health Econ **2007**; 16(10): 1009-23.

2. Barton P, Bryan S, Robinson S. Modelling in the economic evaluation of health care: selecting the appropriate approach. J Health Serv Res Policy **2004**; 9(2): 110-8.

3. Brennan A, Chick SE, Davies R. A taxonomy of model structures for economic evaluation of health technologies. Health Econ **2006**; 15(12): 1295-310.

4. Briggs A, Sculpher M, Claxton K. Decision modelling for health economic evaluation: Oxford university press, **2006**.

5. Moher D, Tsertsvadze A, Tricco AC, et al. A systematic review identified few methods and strategies describing when and how to update systematic reviews. J Clin Epidemiol **2007**; 60(11): 1095-104.

6. Choko AT, MacPherson P, Webb EL, et al. Uptake, Accuracy, Safety, and Linkage into Care over Two Years of Promoting Annual Self-Testing for HIV in Blantyre, Malawi: A Community-Based Prospective Study. PLoS Med **2015**; 12(9): e1001873.

7. MacPherson P, Corbett EL, Makombe SD, et al. Determinants and consequences of failure of linkage to antiretroviral therapy at primary care level in Blantyre, Malawi: a prospective cohort study. PLoS One **2012**; 7(9): e44794.

8. Rosen S, Fox MP. Retention in HIV Care between Testing and Treatment in Sub-Saharan Africa: A Systematic Review. PLoS Med **2011**; 8(7): e1001056.

9. Choko AT, MacPherson P, Webb EL, et al. Uptake, accuracy, safety and linkage into care over two years of promoting annual self-testing for HIV in a community-based prospective study in Blantyre, Malawi. Plos Med (in press) **2015**.

10. Granich RM, Gilks CF, Dye C, De Cock KM, Williams BG. Universal voluntary HIV testing with immediate antiretroviral therapy as a strategy for elimination of HIV transmission: a mathematical model. Lancet **2009**; 373(9657): 48-57.

11. Tanser F, Barnighausen T, Grapsa E, Zaidi J, Newell ML. High coverage of ART associated with decline in risk of HIV acquisition in rural KwaZulu-Natal, South Africa. Science **2013**; 339(6122): 966-71.

12. WHO. Global Health Observatory data repository. Available at: <http://apps.who.int/gho/data/?theme=main&vid=60980>

13. Anglaret X, Minga A, Gabillard D, et al. AIDS and non-AIDS morbidity and mortality across the spectrum of CD4 cell counts in HIV-infected adults before starting antiretroviral therapy in Cote d'Ivoire. Clin Infect Dis **2012**; 54(5): 714-23.

14. Badri M, Lawn SD, Wood R. Short-term risk of AIDS or death in people infected with HIV-1 before antiretroviral therapy in South Africa: a longitudinal study. Lancet **2006**; 368(9543): 1254-9.

15. Jaffar S, Grant AD, Whitworth J, Smith PG, Whittle H. The natural history of HIV-1 and HIV-2 infections in adults in Africa: a literature review. Bull World Health Organ **2004**; 82(6): 462-9.

16. Geng EH, Bwana MB, Muyindike W, et al. Failure to initiate antiretroviral therapy, loss to follow-up and mortality among HIV-infected patients during the pre-ART period in Uganda. J Acquir Immune Defic Syndr **2013**; 63(2): e64-71.

17. IeDEA. International Epidemiologic Databases to Evaluate AIDS. Web link: <http://www.iedea.org> **no date**.

18. Hoffmann CJ, Schomaker M, Fox MP, et al. CD4 count slope and mortality in HIV-infected patients on antiretroviral therapy: multicohort analysis from South Africa. J Acquir Immune Defic Syndr **2013**; 63(1): 34-41.

19. Holmes CB, Wood R, Badri M, et al. CD4 decline and incidence of opportunistic infections in Cape Town, South Africa: implications for prophylaxis and treatment. J Acquir Immune Defic Syndr **2006**; 42(4): 464-9.

20. Badri M, Maartens G, Mandalia S, et al. Cost-effectiveness of highly active antiretroviral therapy in South Africa. PLoS Med **2006**; 3(1): e4.

21. Williams BG, Korenromp EL, Gouws E, Schmid GP, Auvert B, Dye C. HIV infection, antiretroviral therapy, and CD4+ cell count distributions in African populations. J Infect Dis **2006**; 194(10): 1450-8.

22. Mboto CI, Davies-Russell A, Fielder M, Jewell AP. CD4+ lymphocyte values and trends in individuals infected with human immunodeficiency virus and/or co-infected with hepatitis C virus in the Gambia. Afr Health Sci **2009**; 9(3): 130-6.

23. Katubulushi M, Zulu I, Yavwa F, Kelly P. Slow decline in CD4 cell count in a cohort of HIV-infected adults living in Lusaka, Zambia. AIDS **2005**; 19(1): 102-3.

24. Lodi S, Phillips A, Touloumi G, et al. Time from human immunodeficiency virus seroconversion to reaching CD4+ cell count thresholds <200, <350, and <500 Cells/mm(3): assessment of need following changes in treatment guidelines. Clin Infect Dis **2011**; 53(8): 817-25.

25. Martinson NA, Gupte N, Msandiwa R, et al. CD4 and viral load dynamics in antiretroviral-naive HIV-infected adults from Soweto, South Africa: a prospective cohort. PLoS One **2014**; 9(5): e96369.

26. May M, Wood R, Myer L, et al. CD4(+) T cell count decreases by ethnicity among untreated patients with HIV infection in South Africa and Switzerland. J Infect Dis **2009**; 200(11): 1729-35.

27. Lessells RJ, Mutevedzi PC, Cooke GS, Newell ML. Retention in HIV care for individuals not yet eligible for antiretroviral therapy: rural KwaZulu-Natal, South Africa. J Acquir Immune Defic Syndr **2011**; 56(3): e79-86.

28. Plazy M, Orne-Gliemann J, Dabis F, Dray-Spira R. Retention in care prior to antiretroviral treatment eligibility in sub-Saharan Africa: a systematic review of the literature. BMJ Open **2015**; 5(6): e006927.

29. Clouse K, Pettifor AE, Maskew M, et al. Patient retention from HIV diagnosis through one year on antiretroviral therapy at a primary health care clinic in Johannesburg, South Africa. J Acquir Immune Defic Syndr **2013**; 62(2): e39-46.

30. Hassan AS, Fielding KL, Thuo NM, Nabwera HM, Sanders EJ, Berkley JA. Early loss to follow-up of recently diagnosed HIV-infected adults from routine pre-ART care in a rural district hospital in Kenya: a cohort study. Trop Med Int Health **2012**; 17(1): 82-93.

31. Honge BL, Jespersen S, Nordentoft PB, et al. Loss to follow-up occurs at all stages in the diagnostic and follow-up period among HIV-infected patients in Guinea-Bissau: a 7-year retrospective cohort study. BMJ Open **2013**; 3(10): e003499.

32. Kranzer K, Zeinecker J, Ginsberg P, et al. Linkage to HIV care and antiretroviral therapy in Cape Town, South Africa. PLoS One **2010**; 5(11): e13801.

33. Namusobya J, Semitala FC, Amanyire G, et al. High retention in care among HIV-infected patients entering care with CD4 levels >350 cells/muL under routine program conditions in Uganda. Clin Infect Dis **2013**; 57(9): 1343-50.

34. Larson BA, Brennan A, McNamara L, et al. Early loss to follow up after enrolment in pre-ART care at a large public clinic in Johannesburg, South Africa. Trop Med Int Health **2010**; 15 Suppl 1: 43-7.

35. Fox MP, Rosen S. Patient retention in antiretroviral therapy programs up to three years on treatment in sub-Saharan Africa, 2007-2009: systematic review. Trop Med Int Health **2010**; 15 Suppl 1: 1-15.

36. Maheswaran H, Petrou S, MacPherson P, et al. Economic costs and health-related quality of life outcomes of HIV treatment following self- and facility-based HIV testing in a cluster randomised trial. J Acquir Immune Defic Syndr **2017**.

37. Maheswaran H, Petrou S, Cohen D, et al. Economic costs and health-related quality of life outcomes of hospitalised patients with high HIV prevalence: A prospective hospital cohort study in Malawi. PLoS One (under review) **2017**.

38. Eddy DM, Hollingworth W, Caro JJ, et al. Model transparency and validation: a report of the ISPOR-SMDM Modeling Good Research Practices Task Force--7. Value Health **2012**; 15(6): 843-50.

39. Choko AT, Desmond N, Webb EL, et al. The Uptake and Accuracy of Oral Kits for HIV Self-Testing in High HIV Prevalence Setting: A Cross-Sectional Feasibility Study in Blantyre, Malawi. PLoS Medicine **2011**; 8(10): e1001102.

40. Macpherson P, Lalloo DG, Choko AT, et al. Suboptimal patterns of provider initiated HIV testing and counselling, antiretroviral therapy eligibility assessment and referral in primary health clinic attendees in Blantyre, Malawi*. Trop Med Int Health **2012**.

41. Faal M, Naidoo N, Glencross DK, Venter WD, Osih R. Providing immediate CD4 count results at HIV testing improves ART initiation. J Acquir Immune Defic Syndr **2011**; 58(3): e54-9.

42. May M, Boulle A, Phiri S, et al. Prognosis of patients with HIV-1 infection starting antiretroviral therapy in sub-Saharan Africa: a collaborative analysis of scale-up programmes. Lancet **2010**; 376(9739): 449-57.
